# Adaptive single-KIR^+^NKG2C^+^ NK cells expanded from select superdonors show potent missing-self reactivity and efficiently control HLA-mismatched acute myeloid leukemia

**DOI:** 10.1136/jitc-2022-005577

**Published:** 2022-11-01

**Authors:** Alvaro Haroun-Izquierdo, Marianna Vincenti, Herman Netskar, Hanna van Ooijen, Bin Zhang, Laura Bendzick, Minoru Kanaya, Pouria Momayyezi, Shuo Li, Merete Thune Wiiger, Hanna Julie Hoel, Silje Zandstra Krokeide, Veronika Kremer, Geir Tjonnfjord, Stéphanie Berggren, Kristina Wikström, Pontus Blomberg, Evren Alici, Martin Felices, Björn Önfelt, Petter Höglund, Bahram Valamehr, Hans-Gustaf Ljunggren, Andreas Björklund, Quirin Hammer, Lise Kveberg, Frank Cichocki, Jeffrey S Miller, Karl-Johan Malmberg, Ebba Sohlberg

**Affiliations:** 1 Center for Infectious Medicine, Department of Medicine Huddinge, Karolinska Institutet, Stockholm, Sweden; 2 Department of Cancer Immunology, Institute for Cancer Research, Oslo University Hospital and University of Oslo, Oslo, Norway; 3 Department of Applied Physics, Science for Life Laboratory, KTH Royal Institute of Technology, Stockholm, Sweden; 4 University of Minnesota, Masonic Cancer Center, Minneapolis, Minnesota, USA; 5 Department of Hematology, Oslo University Hospital and K.G. Jebsen Centre for B-cell malignancies, Institute of Clinical Medicine, University of Oslo, Oslo, Norway; 6 Vecura, Karolinska Center for Cell Therapy Clinical Research Center, Karolinska University Hospital, Stockholm, Sweden; 7 Center for Hematology and Regenerative Medicine, Department of Medicine Huddinge, Karolinska Institutet, Stockholm, Sweden; 8 Department of Microbiology, Tumour and Cell Biology, Karolinska Institutet, Stockholm, Sweden; 9 Department of Clinical Immunology and Transfusion Medicine, Karolinska University Hospital, Stockholm, Sweden; 10 Fate Therapeutics Inc, La Jolla, California, USA; 11 Department of Cellular Therapy and Allogeneic Stem Cell Transplantation, Karolinska University Hospital, Stockholm, Sweden

**Keywords:** killer cells, natural, immunotherapy, adoptive, immunity, innate

## Abstract

**Background:**

Natural killer (NK) cells hold great promise as a source for allogeneic cell therapy against hematological malignancies, including acute myeloid leukemia (AML). Current treatments are hampered by variability in NK cell subset responses, a limitation which could be circumvented by specific expansion of highly potent single killer immunoglobulin-like receptor (KIR)^+^NKG2C^+^ adaptive NK cells to maximize missing-self reactivity.

**Methods:**

We developed a GMP-compliant protocol to expand adaptive NK cells from cryopreserved cells derived from select third-party superdonors, that is, donors harboring large adaptive NK cell subsets with desired KIR specificities at baseline. We studied the adaptive state of the cell product (ADAPT-NK) by flow cytometry and mass cytometry as well as cellular indexing of transcriptomes and epitopes by sequencing (CITE-Seq). We investigated the functional responses of ADAPT-NK cells against a wide range of tumor target cell lines and primary AML samples using flow cytometry and IncuCyte as well as in a mouse model of AML.

**Results:**

ADAPT-NK cells were >90% pure with a homogeneous expression of a single self-HLA specific KIR and expanded a median of 470-fold. The ADAPT-NK cells largely retained their adaptive transcriptional signature with activation of effector programs without signs of exhaustion. ADAPT-NK cells showed high degranulation capacity and efficient killing of HLA-C/KIR mismatched tumor cell lines as well as primary leukemic blasts from AML patients. Finally, the expanded adaptive NK cells had preserved robust antibody-dependent cellular cytotoxicity potential and combination of ADAPT-NK cells with an anti-CD16/IL-15/anti-CD33 tri-specific engager led to near-complete killing of resistant CD45^dim^ blast subtypes.

**Conclusions:**

These preclinical data demonstrate the feasibility of off-the-shelf therapy with a non-engineered, yet highly specific, NK cell population with full missing-self recognition capability.

**WHAT IS ALREADY KNOWN ON THIS TOPIC**

- Adoptive natural killer (NK) cell therapy in combination with hematopoietic stem cell transplantation can provide cure for acute myeloid leukemia (AML) but is in part dependent on variable NK cell reactivity. Adaptive NK cells are a highly potent NK cell subset that can be used to maximize ‘missing-self’ reactivity against tumor cells.

**WHAT THIS STUDY ADDS**

- It describes a novel GMP-compliant protocol to expand clinically relevant numbers of single self-killer immunoglobulin-like receptor^+^ adaptive NK cells from third-party ‘superdonors’ that provide strong alloreactivity in a mouse model of AML as well as against primary AML blasts ex vivo.

**HOW THIS STUDY MIGHT AFFECT RESEARCH, PRACTICE OR POLICY**

- These preclinical data demonstrate the feasibility of cell therapy with a non-engineered and yet highly specific NK cell population with maximized missing-self recognition.

## Introduction

Natural killer (NK) cells can mediate strong antitumor immunity, which is being explored in immunotherapy strategies for human malignancies. Adoptive transfer of allogeneic NK cells has a good safety profile without cytokine release syndrome, neurotoxicity, or GvH disease and has shown efficacy for subgroups of refractory leukemia patients.^1–5^ Current approaches to generate NK cells for therapy include sourcing and expanding NK cell lines, donor-blood and cord-blood NK cells, and inducible pluripotent stem cell (iPSC)-derived NK cells using feeder cell lines and cytokines.^6^ Despite promising results, the persistence, and variable antitumor responses of different NK cell subsets along with high diversity of NK cell repertoires in healthy donors,^7^ represents a potential limitation of donor-derived NK cell-based therapy strategies. Identifying biology-driven approaches that can enhance these attributes prior to adoptive transfer and establishing selection procedures for optimal donors therefore represent key bottlenecks in the field.

Early observations showed that NK cell cytotoxicity was triggered by tumor cells that lacked expression of some or all self-MHC class I molecules, referred to as ‘missing-self’ recognition.^8^ Therefore, it was postulated that NK cells might be particularly effective when transferred across HLA barriers,^9 10^ where NK cell alloreactivity would be unleashed through mismatch between donor NK clones bearing inhibitory killer immunoglobulin-like receptor (KIR) specific for self-HLA class I molecules (‘self-KIR’) and recipient cells lacking the cognate HLA class I ligands. For such an NK cell-mediated graft-versus leukemia (GvL) effect to take place, there must be a mismatch in any of the three major KIR-KIR ligand (KIR-L) settings (KIR2DL1 missing HLA-C2, KIR2DL3 missing HLA-C1, or KIR3DL1 missing HLA-Bw4). The importance of NK cell alloreactivity was elegantly explored in landmark studies by the Velardi group, where the size of the alloreactive NK subset was linked to improved outcomes in patients, both in hematopoietic stem cell transplantation (HSCT) and later in adoptive NK cell transfer in acute myeloid leukemia (AML).^2 10 11^ Studies investigating a KIR-L mismatch effect have not always been conclusive though, which may in part be related to the variable frequency of the alloreactive NK cell subset,^12^ in turn due to the stochastic expression of KIR.^13^ Therefore, selectively expanding the alloreactive subset prior to adoptive NK cell transfer to increase missing-self reactivity^14^ would be greatly desired and could lead to clinical benefits.

NK cell differentiation spans from naive CD56^bright^ cells to highly differentiated CD56^dim^ adaptive NK cells induced by cytomegalovirus (CMV) infection.^15 16^ Adaptive NK cells are characterized by the expression of the activating receptor CD94/NKG2C (NKG2C), whose cognate ligand HLA-E also binds to the inhibitory receptor CD94/NKG2A (NKG2A). Engagement of NKG2C by peptide-stabilized HLA-E can elicit effector functions and support adaptive NK cell expansion in vitro.^17–20^ Apart from NKG2C, the adaptive NK cell signature is characterized by a unique surface phenotype, distinct patterns of transcription factors and signaling molecules as well as altered DNA methylation patterns.^21–23^ Adaptive NK cells have a high effector molecule content and an increased capacity for IFN-γ production and antibody-dependent cellular cytotoxicity (ADCC).^18 21 24 25^ Intriguingly, their unique and preferential expression of a single self-KIR^18^ make them especially attractive for strategies aiming at exploiting missing-self recognition since they will demonstrate predictable alloreactivity in HLA-mismatched settings. In allogeneic HSCT, reactivation of CMV and concurrent expansion of adaptive NK cells has been associated with remarkable reductions in AML relapse rates,^26–29^ highlighting their clinical relevance and potential.

We have previously developed an HLA-E feeder-based platform for selective expansion of adaptive NK cells, leading to highly pure populations with potent alloreactivity against primary acute lymphoid leukemia.^30^ Here, we used CD3/CD19-depleted cryopreserved cell material from ‘superdonors’ that harbored large NKG2C^+^ adaptive NK cell subsets with single self-KIR expression, to generate high expansion rates of adaptive NK cells. ADAPT-NK cells largely maintained their adaptive signature, and demonstrated strong alloreactivity against tumor cell lines, in an in vivo AML model and against primary AML samples. Finally, we show that a combination of ADAPT-NK cells with an immune engager enhances reactivity against more resistant AML blast subtypes, broadening the therapeutic utility of the ADAPT-NK cell platform.

## Methods

Additional Methods can be found in online supplemental materials.

10.1136/jitc-2022-005577.supp1 Supplementary data

### Human participants and cell processing

Healthy CMV-seropositive blood donors with large pre-existing adaptive NK cell subpopulations (>20% of NKG2C^+^ with single self-KIR2DL1 or KIR2DL3 expression, denoted as ‘superdonors’) were included in the study. Peripheral blood monunuclear cells (PBMCs) were isolated from buffy coats using Lymphoprep and CD3/CD19-depleted as per manufacturer’s instructions (Miltenyi Biotec). PBMC were also obtained from leukapheresis products that were subsequently CD3/CD19-depleted by CliniMacs (Miltenyi). Depleted cell products were cryopreserved in 90% heat-inactivated fetal bovine serum (FBS, Sigma-Aldrich) or 90% autologous plasma with 10% DMSO until use. To determine donor HLA-C type, DNA was isolated using a commercial kit (DNeasy Blood & Tissue, Qiagen) and the KIR HLA ligand kit was used (Olerup SSP). CMV serology was determined using an ELISA-based assay on plasma obtained during sample preparation. Purified nuclear CMV antigen (AD 169) was used, and the cut-off level for seropositivity was an absorbance of ≥0.2 at a dilution of 1/100.

### ADAPT-NK cell expansion protocol

CD3/CD19-depleted PBMC were co-cultured with 100 or 200 Gy irradiated K562 feeder cells transfected with lentiviral construct to express high levels of HLA-E with an HLA-G-leader-derived peptide (Fate Therapeutics, details in ‘Genetic cell engineering’) at a 1:2 ratio in G-Rex24 plates (Wilson Wolf) at 0.5×10^6^ total cells/cm^2^. Cells were cultured in GMP-grade Stem Cell Growth Medium (CellGenix) supplemented with 10% human ab serum (TCS Biosciences or Access Biologicals), 2mM L-glutamine (Cytiva -FisherScientific) and 100 IU/mL human recombinant IL-2 (Proleukin) for 11 days with 60% medium exchange on day 7, and IL-2 addition days 4, 7 and 10.

### Flow cytometry and mass cytometry

NK cell phenotype and function was evaluated using standard protocols for flow- and mass cytometry. Samples were acquired on a BD LSRII, or LSR-Fortessa 18-color flow cytometer (BD Biosciences) or on a Helios CyTOF instrument (Fluidigm), and data were analyzed with FlowJo software V.10 (BD Biosciences) and R (R Core Team, 2019). Details are available in online supplemental materials including flow cytometry antibodies (online supplemental sTable 1) and mass cytometry antibodies (online supplemental sTable 2).

### Cellular indexing of transcriptomes and epitopes by sequencing CITE-Seq

CITE-seq was performed as outlined in Biolegend ‘TotalSeqTM-A Antibodies and Cell Hashing with 10X Single Cell 3' Reagent Kit V.3 3.1 Protocol’ with minor modifications. Briefly, cells were stained with CD56-biotin (REA 196), followed by TotalSeq antibodies and TotalSeq streptavidin-PE and Live/Dead Aqua (Invitrogen) and subsequently sorted for viable CD56^+^ cells by flow cytometry (online supplemental sFigure 2A, B). To distinguish day 0 and day 11 cells, streptavidin-PE conjugated to different oligos were used, and equal number of sorted viable CD56^+^ cells from the two cell preparations were pooled as one sample. This was followed by standard 10X Genomics library preparation and sequencing workflow (Genomics Core Facility OUH/UiO, Oslo, Norway). Sequencing was performed with the recommended read lengths on the NextSeq500 sequencer (Illumina) and detailed description of the analysis of CITE-seq data is available in online supplemental materials. For network analyses, the DifferentialNet database^31^ (integrated data from experimentally detected protein–protein interactions (PPIs), and RNA sequencing gathered by the Genotype-Tissue Expression consortium) was used to generate tissue-specific (whole blood) PPI networks that were visualized with NetworkAnalyst V.3.0. Enrichment analyses were performed in NWA3.0 with the Gene Ontology (Biological Processes) database.

### Tumor cell lines

The K562 cell line (chronic myeloid leukemia) and 721.221 (EBV-transformed B cell line) were obtained from ATCC (American Type Culture Collection). HL-60 (AML) was obtained from the Miller lab. PANC-1 (Pancreatic ductal adenocarcinoma) and A549 (lung adenocarcinoma) were obtained from Fate Therapeutics. K562, A549 and PANC-1 along with NALM-6 (B cell acute lymphoblastic leukemia) and BJAB (Burkitt-like Lymphoma) were reauthenticated using STR fingerprinting (ATCC).

### AML patient samples

EDTA blood samples were collected at diagnosis from AML patients (Dep. of Hematology, Oslo University Hospital, Oslo, Norway). PBMC were isolated by Lymphoprep and cryopreserved in RPMI 1640 with 60% FBS and 10% DMSO. Patients were genotyped by Illumina NGS platform (Dep. of Immunology, Oslo University Hospital, Oslo, Norway) and for this study, samples were selected from patients of either HLA-C1/C1 or HLA-C2/C2 haplotypes.

### Flow cytometry-based NK cell functional assays

NK cell degranulation and cytokine production was evaluated by mixing ADAPT-NK products at different effector to target (E:T) ratios with tumor targets in 6 hour co-cultures. To measure ADCC, anti-CD20 (MabThera, 1 µg/mL) was added to co-cultures with 721.221 cells. Phorbol-12-myristate-13-acetate (50 ng/mL) + ionomycin (1 µg/mL) (Sigma) was used as positive control. In cytotoxicity assays, target cells were pre-stained with CellTrace Violet (Invitrogen), and FITC-DEVD-FMK (Abcam) to detect active Caspase-3, was added at the start of the incubation. Dead/dying cells were defined as CellTrace^+^ Caspase-3^+^ and/or dead cell marker^+^ and specific cytotoxicity was calculated as: (*% dead ^experimental^- % dead ^target only^*) ÷ (100*% % dead ^target only^*) x 100%. For competitive cytotoxicity assays, K562 expressing an HLA-C1-or HLA-C2-dimer were stained with two different concentrations of CellTrace Violet prior to being mixed at a 1:1 ratio and subsequently seeded at different E:T ratios with ADAPT-NK cells. Cytokine stimulations with IL-12 (10 ng/mL) and IL-18 (10 ng/mL) (both Biotechne) were performed for 25 hours. Further experimental details are provided in online supplemental methods.

### Genetic cell engineering

Engineered cell lines used in this study are described in detail in online supplemental material. Briefly, for the expansion of ADAPT-NK cells, K562 cells (ATCC Cat: CCL-243) were engineered by Fate Therapeutics using a third-generation lentiviral transfer plasmid designed to contain a chimeric protein with the HLA-G leader peptide (1-24) and the mature HLA-E*0103 (22-358) driven by an EF1α promoter. For additional HLA-C and HLA-E variants, K562 were engineered in-house using VSV-G-pseudotyped lentiviral particles with a mammalian LeGO-G2 expression vector, to express the following synthetic proteins: β2m–HLA-C1 (HLA-C*07:01) single chain dimer (HLA-C1-dimer); β2m–HLA-C2 (HLA-C*04:01) single chain dimer (HLA-C2-dimer) and HLA-G_3-11_–β2m–HLA-E*01:01 single chain trimer (HLA-E-trimer). For HLA-E knock-out in NALM-6 cells, Cas9 and a pool of synthetic guide RNAs (sgRNA) (CRISPRevolution sgRNA EZ Kit, Synthego) were used. Single cells were sorted on a FACS Aria II (BD Biosciences) to establish multiple clones. DNA sequencing and flow cytometry confirmed HLA-E depletion in the selected clones used in the study. HLA-E sgRNAs further targeted the HLA-C locus resulting in a combined KO for NALM-6.

### Serial killing assays

The cytotoxic potential of individual NK cells was evaluated using a previously described microwell chip screening assay.^32^ Briefly, labeled NK cells and labeled target cells were seeded in a microwell chip containing complete RPMI supplemented with Sytox Green (Thermo Fisher). Cells stochastically distributed in the 8064 60-μm-wide wells at an average E:T ratio of 1:5. The co-cultures were imaged every 3 hours for 15 hours using a LSM 880 (Carl Zeiss AG) inverted confocal microscope. The images were processed using a custom-built MATLAB script whereby live and dead cells were quantified at all time points, and for each condition a minimum of 500 wells containing a single NK cell at start along with at least 4 live target cells, were included for analysis. Further details are available in online supplemental material.

### IncuCyte measurement of tumor killing

Tumor killing was measured in real-time using the IncuCyte S3 platform. Detailed description of the protocol is available in online supplemental material. Briefly, target cells stably expressing NucLight Red (Essen Biosciences) were overnight rested and subsequently co-cultured with ADAPT-NK cells at different E:T ratios. Images (3/well) from at least two technical replicates for each condition were acquired every 90 min for 48 hours, using a ×10 objective lens and analyzed by IncuCyte Controller v2020A (Essen Biosciences). Graphed readouts represent percentage live target cells.

### In vivo AML tumor model

Detailed description of the protocol is available in online supplemental material. Briefly, NOD.Cg-PrkdcscidIl2rgtm1Wjl/SzJ mice (NSG, Jackson Laboratories) mice were injected intravenously with HL-60 cells stably expressing firefly luciferase (1.5×10^6^/mouse). Four control mice on the same background received no injections. After allowing tumors to engraft for 4 days, bioluminescence imaging was performed, and mice were randomized into three groups: tumor alone, HLA-C/KIR matched ADAPT-NK cells (flat-dose 5×10^6^/mouse) or HLA-C/KIR mismatched ADAPT-NK cells (flat-dose 5×10^6^/mouse). Mice receiving NK cell injections were also injected intraperitoneally with IL-15 (National Cancer Institute, 6 ug/mouse) twice weekly for 3 weeks. Bioluminescence imaging was performed weekly to track tumor burden using the IVIS Spectrum In Vivo Imaging System (Perkin-Elmer).

### Statistical analyses

Statistical analyses and visualization were performed using Prism V.9 (GraphPad) and R with the *ggplot2* and *dunn.test* packages. Paired Student’s t-tests were used for paired comparisons. Repeated measures one-way analysis of variance (ANOVA) with Tukey’s correction was used to analyze three groups (flow, mass- cytometry and CITE-Seq). One-way ANOVA with Šidák’s multiple comparison correction was performed for NK cytotoxicity assays evaluated by flow cytometry and in the IncuCyte platform. Two-way ANOVA with Tukey’s multiple comparison correction was performed to analyze the in vivo AML tumor model. Nonparametric Spearman-rank correlation was performed for evaluation of HLA-E in AML patient samples and NK cell cytotoxicity against these.

## Results

### Efficient expansion of single-self-KIR^+^NKG2C^+^ adaptive NK cells (ADAPT-NK)

**Figure 1:**
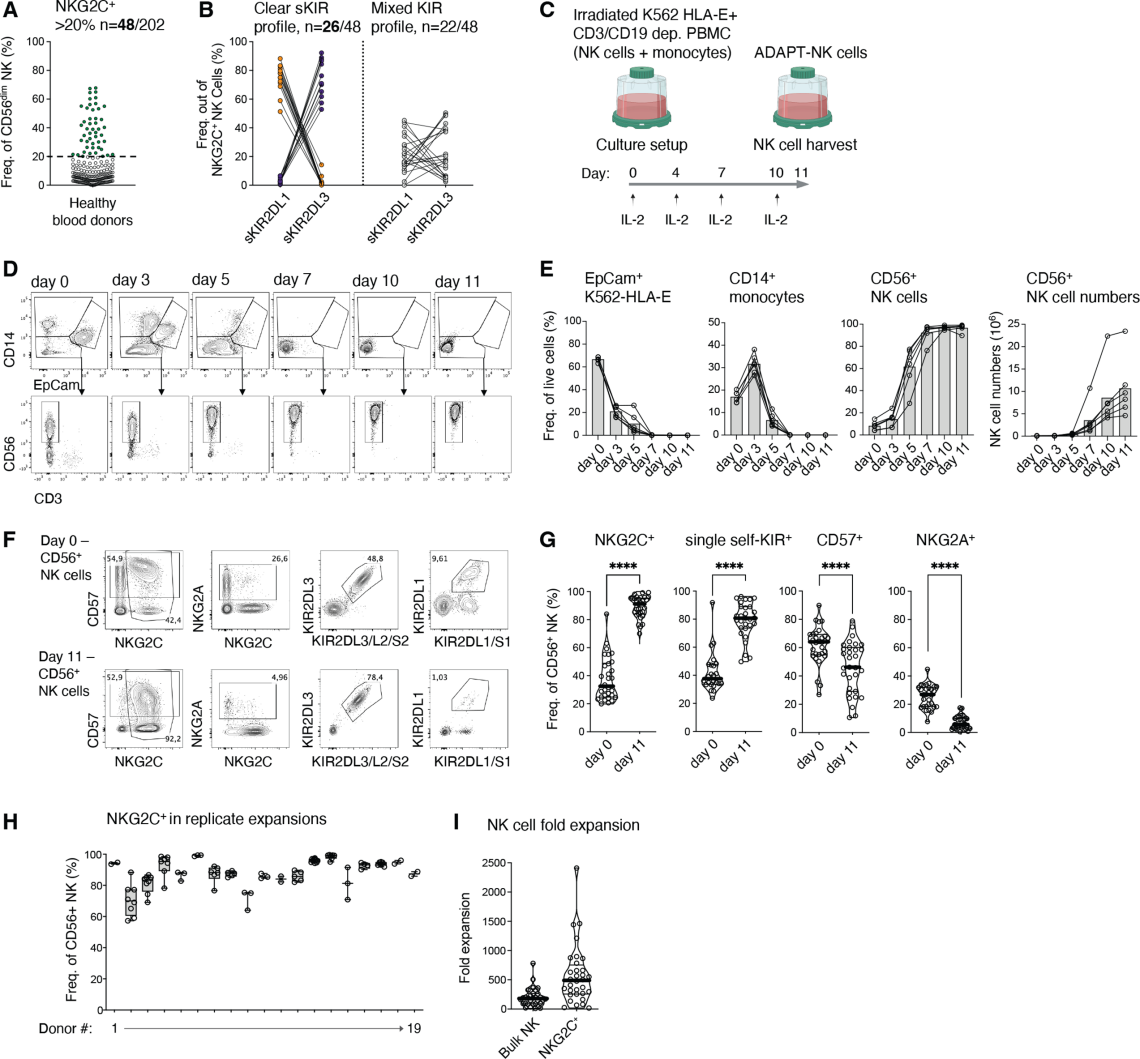
Selective expansion of single-self KIR^+^NKG2C^+^ adaptive NK cells. (A) NKG2C^+^ out of CD56^dim^ NK cells in a cohort of 202 healthy blood donors and in (B) KIR2DL1^+^KIR2DL3^-^ or KIR2DL1^-^KIR2DL3^+^ out of NKG2C^+^CD56^dim^ NK cells in the 48 healthy blood donors with over >20% NKG2C^+^ NK cell subsets. (C) ADAPT-NK protocol design. Flow cytometry analysis of (D, E) EpCam^+^ K562-HLA-E feeders, CD14^+^ monocytes and CD56^+^ NK cells on days 0–11 and (F, G) NKG2C^+^, KIR2DL1^+^ KIR2DL3^-^ or KIR2DL1^-^ KIR2DL3^+^ (‘single self-KIR’), CD57^+^ and NKG2A^+^ among total CD56^+^ NK cells on day 0 and day 11. (H) NKG2C^+^ frequencies on day 11 where each dot represents a separate expansion of the same donor material. (I) Fold expansion in cell numbers of total and NKG2C^+^ ADAPT-NK cells on day 11. (A), n=202, (B), n= 48,) (E), n=6, (G-I), n=27–31, in G and I the median of each donor from 34 independent experiments is shown. (H) n=19 from 2–10 independent experiments. Statistical differences were tested using paired t-tests, *p<0.05, **p<0.01, ***p<0.001, ****p<0.0001. KIR, killer immunoglobulin-like receptor; NK, natural killer; PBMC, peripheral blood monunuclar cells.

NKG2C^+^ adaptive NK cells tend to express only a single self-KIR, a fact that translates to high alloreactive potential if transferred to recipients with KIR-L (HLA-C) mismatch.^18 30^ Re-examining data from our previously published cohort of healthy blood donors,^18^ 48/202 donors had a large (over 20%) NKG2C^+^ adaptive NK cell subset, and of those, 26 had a clear single-self KIR2DL1^+^KIR2DL3^-^ or KIR2DL1^-^KIR2DL3^+^ expression profile (figure 1A, B) and were subsequently denoted as ‘superdonors’. Based on CD3/CD19-depleted material from these select superdonors, a GMP-compliant protocol to expand adaptive NK cells was developed (figure 1C). Monocytes and feeder cells were undetectable in the co-cultures by day 7, concomitant with a rise in NK cell numbers (figure 1D, E). On day 11, a median of 91% of NK cells were NKG2C^+^ and 81% had single-self-KIR expression (either KIR2DL1^+^KIR2DL3^-^ or KIR2DL1^-^KIR2DL3^+^, figure 1F, G). Hereafter, we refer to this expanded adaptive NK cell product as ‘ADAPT-NK’ cells. While CD57 expression declined slightly, less than 20% of cells expressed NKG2A post-expansion (figure 1F, G). Replicate expansions of cell material obtained from the same donor and from several donations, gave stable results (median 88% NKG2C^+^, SD 3.64% figure 1H). We achieved a median of 470-fold expansion of adaptive NK cells with some donors displaying over 1000-fold expansion rates (figure 1I). Thus, the developed expansion protocol produced high fold changes of single self-KIR^+^NKG2C^+^ adaptive NK cells in 11 days, opening an avenue for clinical development.

### ADAPT-NK cells retain an adaptive transcriptional signature with activation of effector programs and without signs of exhaustion

**Figure 2:**
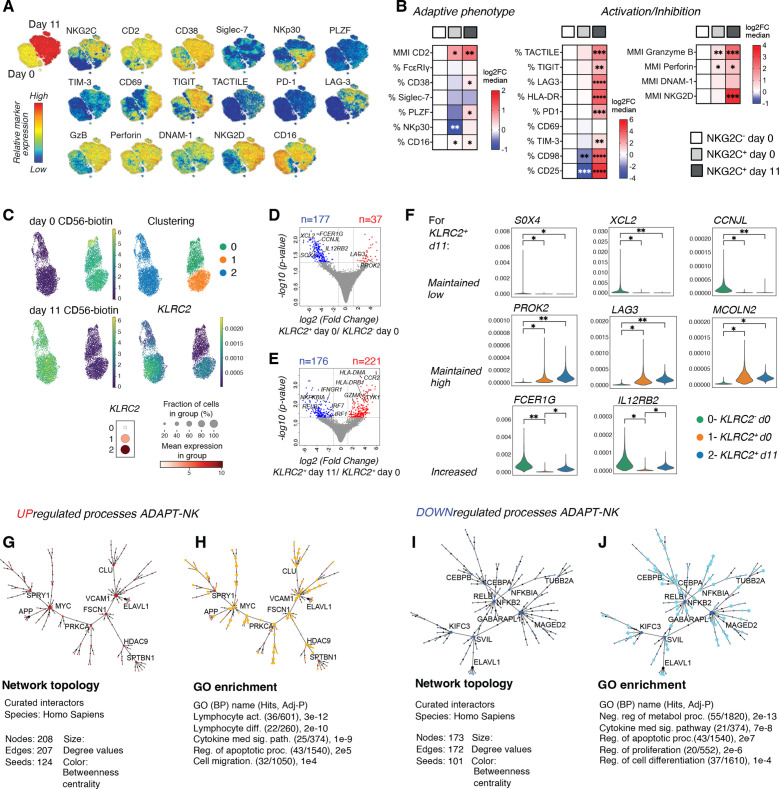
ADAPT-NK cells show a retained adaptive transcriptional signature with activation of effector programs. Extended phenotyping by flow cytometry and mass cytometry as well as CITE-Seq analysis. (A) Relative expression for assessed markers by mass cytometry shown on a t-SNE clustering of sampled events from all individuals (day 0 n=4, day 11 n=6). (B) Frequencies of marker expression or mean metal intensity depicted as median log2 fold change as compared with NKG2C^-^ day 0 from flow- and mass cytometry data. Significances in heatmaps are given as compared with NKG2C^-^ day 0. (C) UMAP embedding of the CITE-seq data colored based on CD56-biotin labeling for day 0 and day 11, cluster assignment (0–2) and expression of KLRC2 in those clusters. Dotplot showing expression of KLRC2 by cluster. (D) 177 downregulated (blue) and 37 upregulated (red) DE genes between KLRC2^+^ day 0 and KLRC2^-^ day 0 and (E) 176 downregulated (blue) and 221 upregulated (red) DE genes between KLRC2^+^ day 0 and KLRC2^+^ day 11 after filtering for log2FC>1.2. (F) Relative gene expression of selected adaptive genes. ‘Maintained’ indicates significance of *KLRC2^+^* day 11 to *KLRC2^-^* day 0 while non-significant to *KLRC2^+^* day 0. (G) Topology and content of the protein-protein interaction (PPI) network driven by upregulated DE genes for day 11 *KLRC2* ^+^ as compared with day 0 *KLRC2* ^+^ NK cells. (H) Gene set enrichment analysis of the Steiner forest PPI network with genes that when identified in GO (BP) processes were highlighted (orange). (I) Topology and content of the PPI Steiner forest network driven by downregulated DE genes for day 11 *KLRC2* ^+^ as compared with day 0 *KLRC2* ^+^ NK cells. (J) GSEA of the PPI network with genes that when identified in GO (BP) processes were highlighted (teal). For flow cytometry and mass cytometry n=4–7, CITE-seq n=1. *p<0.05, **p<0.01, ***p<0.001, ****p<0.0001, in one-way analysis of variance (ANOVA) with Tukey’s correction. BP, biological process; GSEA, gene set enrichment analysis; DE; differentially expressed; GO; gene ontology; GSEA, gene set enrichment analysis; PPI, protein–protein interaction; t-SNE, t-distributed stochastic neighbor embedding; UMAP, uniform manifold approximation and projection.

To assess the adaptive state in NKG2C^+^ NK cells pre-expansion and post-expansion, we performed extended phenotyping by flow cytometr and mass cytometry (figure 2 and online supplemental sFigure 1) as well as cellular indexing of transcriptomes and epitopes by sequencing (CITE-Seq) (figure 2 and online supplemental sFigure 2). For mass cytometry, gating was performed on viable CD56^+^ NK cells and visualized in t-SNE plots, with distinct topological regions corresponding to day 0 and day 11 NK cells (figure 2A and online supplemental sFigure 1A). On day 0, NKG2C^+^ cells displayed low expression of NKp30, Siglec-7, PLZF, CD38 and high expression of CD2 as compared with NKG2C^-^ conventional NK (cNK) cells in accordance with previously published phenotypes.^22 33^ In addition to NKG2C and single-KIR on ADAPT-NK cells, key adaptive markers such as CD16, CD2 and Siglec-7 were similarly expressed post-expansion whereas CD38, NKp30, PLZF and FcεRIγ were upregulated as compared with resting NKG2C^+^ adaptive NK cells (figure 2A, B, significance given in online supplemental sFigure 1C). Further, activation-related TIM-3, HLA-DR, CD25, CD69 and CD98 and inhibitory check point molecules TIGIT, TACTILE, PD-1 and LAG-3 were upregulated. Concomitantly, perforin and DNAM-1 were maintained at a high expression level and granzyme B and NKG2D levels were upregulated (figure 2A, B and online supplemental sFigure 1D).

The transcriptional profile of pre-expansion and postexpansion NK cells was interrogated using CITE-Seq (8013 cells sequenced, online supplemental sFigure 2A, B), revealing distinct clusters corresponding to day 0 *KLRC2* ^-^ cNK and *KLRC2* ^+^ adaptive NK cells as well as day 11 *KLRC2^+^* NK cells (figure 2C). At baseline, *KLRC2^+^* NK had 177 downregulated and 37 upregulated genes as compared with *KLRC2* ^-^ cNK (figure 2D). Post-expansion, *KLRC2* ^+^ ADAPT-NK had 176 downregulated and 221 upregulated genes as compared with baseline *KLRC2* ^+^ adaptive NK (figure 2E). Consistent with published transcriptomes for adaptive NK cells,^23 34^ baseline *KLRC2* ^+^ NK cells had higher expression of *LAG3, PROK2* and *MCOLN2* and lower expression of *FCER1G*, *IL12RB2*, *XCL2*, *CCNJL* and *SOX4* as compared with *KLRC2* ^-^ cNK. Postexpansion, *KLRC2* ^+^ ADAPT-NK cells showed upregulation of *FCER1G* and *IL12RB2* whereas expression of other adaptive signature genes was similar (figure 2F).

Next, the transcriptional signature with 397 differentially expressed (DE) genes for *KLRC2* ^+^ ADAPT-NK cells postexpansion (figure 2E) was interrogated. PPI analysis with the upregulated DE genes as a base resulted in a network of 208 elements (nodes; figure 2G), which was queried for pathway enrichment revealing lymphocyte activation and differentiation as highly upregulated processes (figure 2H, online supplemental sFigure 2C and online supplemental sTable 3). Using the downregulated DE genes as a base resulted in a network of 173 elements (nodes; figure 2I) where regulation of cell metabolism and proliferation dominated (figure 2J, online supplemental sFigure 2D and online supplemental sTable 4). Several DE genes annotated to cytokine-mediated signaling pathway and regulation of apoptosis were discovered among the observed downregulated and upregulated processes. Notably, despite a phenotypic resemblance to exhausted T cells,^35^ cell exhaustion or senescence were not among the enriched processes and the overlap to transcriptional T cell exhaustion signatures was minimal (online supplemental sFigure 2E, F). Taken together, this reveals that expanded ADAPT-NK cells largely retained their adaptive state with evidence of metabolic and effector cell activation without signs of exhaustion.

### ADAPT-NK cells are highly functional with predictable alloreactivity

**Figure 3:**
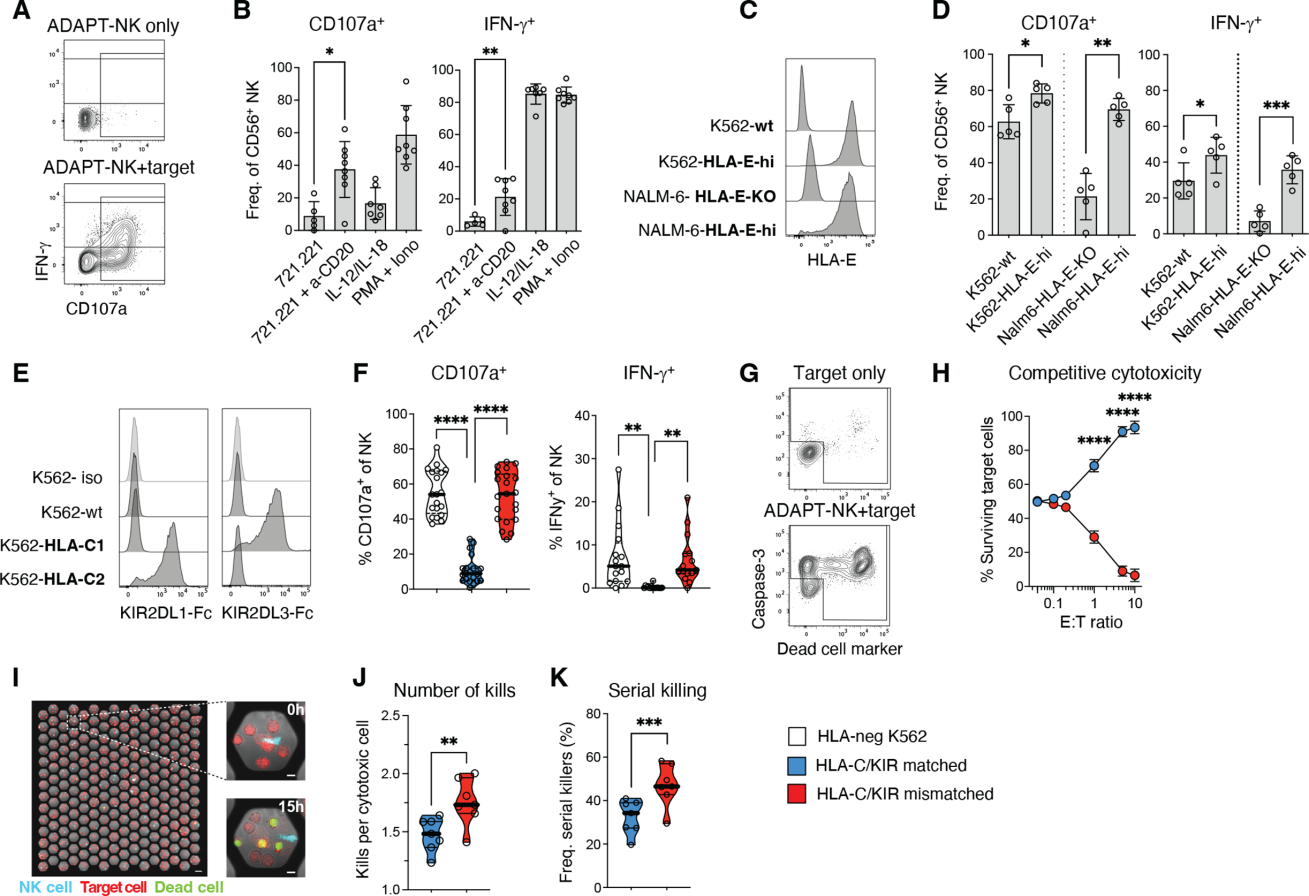
ADAPT-NK cells are highly functional with predictable alloreactivity. (A) Functional analysis of NK cells by flow cytometry in terms of degranulation (CD107a) and IFN-γ production. (B) ADAPT-NK responses to 721.221 target cells with or without anti-CD20 (MabThera) addition, and to IL-12+IL-18 or PMA+Ionomycin stimulation. (C, D) ADAPT-NK responses to K562 or NALM-6 tumor target cells with varying expression levels of HLA-E. (E) Determination of HLA-C expression by means of KIR-Fc staining for K562 engineered with single chain β2m HLA-C1 or HLA-C2 dimers. (F) Responses of KIR2DL1^+^ KIR2DL3^-^ KIR2DL2^-^ NKG2A^-^ or KIR2DL1^-^ KIR2DL3^+^ KIR2DL2^-^ NKG2A^-^ ADAPT-NK cells to HLA-C1 or HLA-C2 K562 in the HLA-C/KIR matched and mismatched setting. (G) Target cell death judged by Dead Cell Marker and caspase-3 staining to determine freq. of remaining live target cells. (H) Frequency of live target cells after mixed target cell assays with ADAPT-NK cells in the HLA-C/KIR matched and mismatched setting. (I–K) The cytotoxic potential of individual NK cells in the HLA-C/KIR matched and mismatched setting was assessed in a microwell screening assay. (J) Average number of kills performed by the cytotoxic NK cells. (K) Corresponding fraction of cytotoxic NK cells killing >1 target. For (B) n=8 in two independent experiments, (D) n=5 in one independent experiment, (F–H) n=11 in three independent experiments with KIR2DL1^+^ and KIR2DL3^+^ subsets analyzed in all donors, (I–K) n=7 in four independent experiments. Statistical differences were calculated using paired t-tests (in B, D, J–K) and a one-way ANOVA with Sidak’s correction (F–H). *p<0.05, **p<0.01, ***p<0.001, p<0.0001. ANOVA, analysis of variance; KIR, killer immunoglobulin-like receptor; NK, natural killer; PMA, phorbol myristate acetate.

To elucidate the functional capacity of the ADAPT-NK cells, we next explored responses to a range of stimuli. We observed strong ADCC against anti-CD20-coated 721.221 B-cell lymphoma targets but also robust IFN-γ production in response to IL-12/IL-18 stimulation (figure 3A, B), suggesting that ADAPT-NK cells retain an adaptive NK cell functional profile and, in addition, become more responsive to cytokine stimulation.^22 26–28^ To investigate the contribution of signaling mediated via NKG2C/HLA-E interactions for ADAPT-NK cell functionality, expanded cells were tested against targets without HLA-E or engineered to express high levels of a β2m HLA-E single-chain trimer with forced presentation of the HLA-G leader peptide. K562 and NALM-6 target cells with high levels of HLA-E trimer elicited strong responses from ADAPT-NK cells above those of K562 wildtype (K562wt) or NALM-6 HLA-E KO, showing that the NKG2C receptor is signaling and contributes to the functional response of ADAPT-NK cells (figure 3C, D).

To test the HLA-Cspecificity of ADAPT-NK responses, expanded NKG2C^+^KIR2DL1^+^KIR2DL3^-^ or KIR2DL3^+^KIR2DL1^-^ ADAPT-NK cells were stimulated with K562wt cells or K562 cells engineered to express high levels of single chain β2m HLA-C1 or HLA-C2 dimers. Strong responses were observed in the HLA-C/KIR mismatched setting but ADAPT-NK cells were inhibited in the matched setting (figure 3E, F and online supplemental sFigure 3A). In mixed target cell assays, ADAPT-NK cells preferentially killed HLA-C mismatched targets (figure 3G, H). Assessing the cytotoxic potential of individual NK cells in a microwell screening assay,^32^ there were significantly more kills per cytotoxic cell in the HLA-C/KIR mismatched setting and enhanced serial killing ability (figure 3I–K). These results revealed that ADAPT-NK cells efficiently convert the HLA-E check point into an activation signal and show specific and predictable alloreactivity against K562 targets expressing high levels of mismatched HLA-C.

### Efficient alloreactivity against tumor cell lines and in vivo efficacy of ADAPT-NK cells

**Figure 4:**
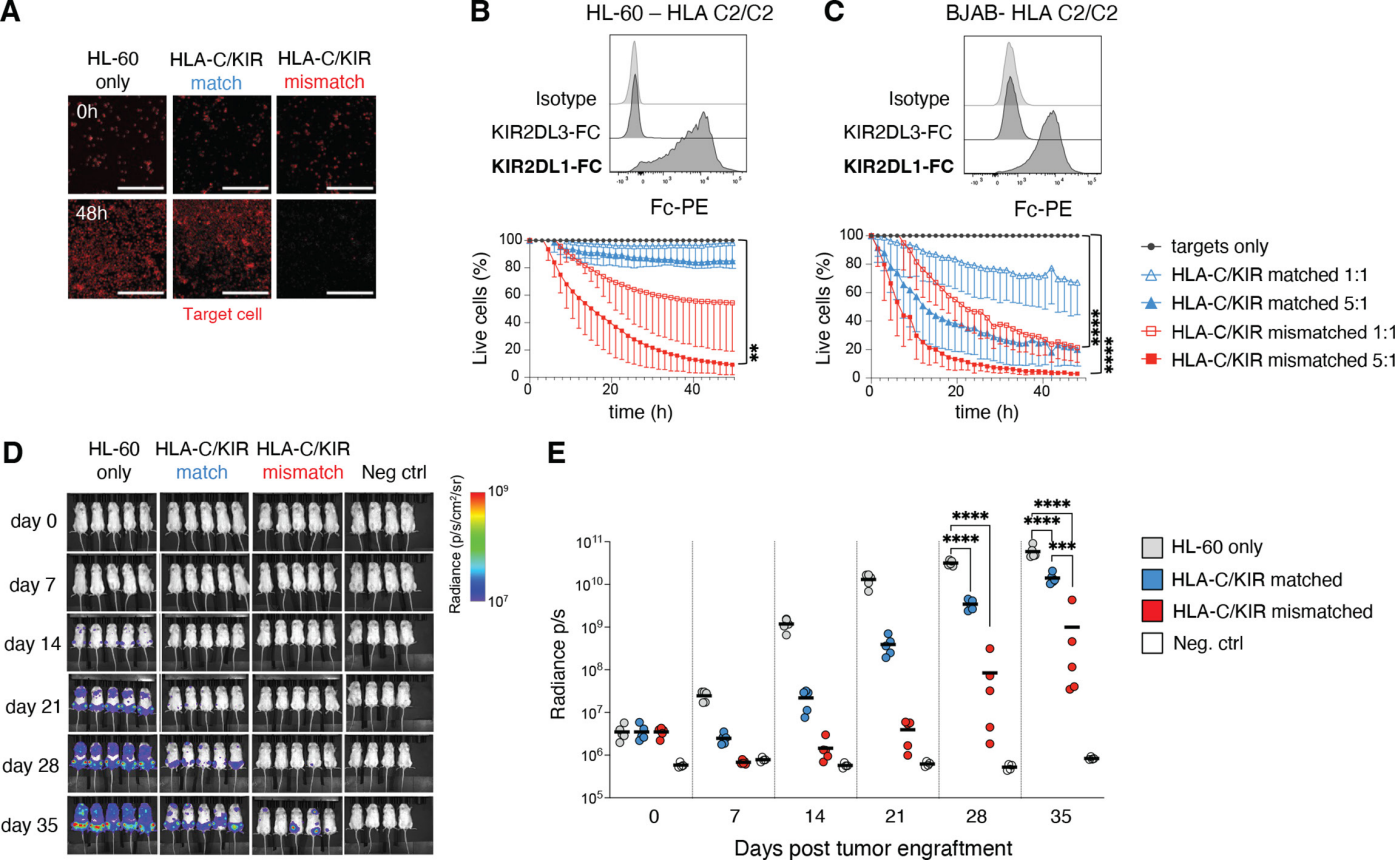
Efficient killing of HLA-C/KIR mismatched tumor cell lines and in vivo efficacy of expanded ADAPT-NK cells. (A–C) Determination of killing ability of ADAPT-NK cells over 48 hours against tumor cell lines of various HLA-C genotypes in the IncuCyte platform, in bold the expected KIR-Fc binding. n=4–9 donors, in at least two independent experiments for each target cell line. Data are displayed as mean (±SD) and significance is given between HLA-C/KIR mismatched and targets only. Scalebar represents 300 µM. (D) a representative example from two independent experiments of bioluminescence in NSG mice injected with luciferase tagged HL-60 4 days prior to injection with a flat-dose of HLA-C/KIR matched or mismatched ADAPT-NK cells and subsequent evaluation for 35 days, compiled data displayed as means in (E). Fifteen mice were used with n=2 ADAPT-NK cells. Four control mice were used. Statistical differences were tested using a one-way ANOVA with Sidak’s correction (B, C) or a two-way ANOVA followed by Tukey’s multiple comparison correction (E). *p<0.05, **p<0.01, ***p<0.001, ****p<0.0001. ANOVA, analysis of variance; KIR, killer immunoglobulin-like receptor; NK, natural killer.

The importance of alloreactivity for tumor targeting was apparent when assessing the ADAPT-NK cell product. We next tested the long-term killing ability against tumor cell lines of various HLA-C genotypes but overall low/negative for HLA-E (figure 4A–C and online supplemental sFigure 3B–F). A group of tumor lines displayed negligible expression of HLA-C and were subsequently killed equally well by HLA-C/KIR-matched and mismatched ADAPT-NK cells (online supplemental sFigure 3C–F). Two HLA-C2/C2 tumor lines, BJAB (Burkitt-like lymphoma) and HL-60 (AML), displayed significant levels of HLA-C2 as assessed by KIR2DL1-Fc binding, and high rates of killing could be observed in the mismatched setting (figure 4A–C).

To evaluate the in vivo impact of ADAPT-NK cell alloreactivity, NSG mice were injected with luciferase-tagged HL-60 cells followed by a flat dose of 5×10^6^ HLA-C/KIR-matched or mismatched ADAPT-NK, under in vivo IL-15 support. The tumor burden was significantly reduced by ADAPT-NK cell transfer for the duration of the experiment, with substantially better leukemia control by HLA-C/KIR mismatched ADAPT-NK cells by day 35 (figure 4D, E). Together this demonstrates the therapeutic efficacy of alloreactive ADAPT-NK against HLA-C mismatched tumor cells and underscores their ability to deliver a maximized missing-self response in settings with high levels of mismatched HLA-C.

### ADAPT-NK cells show potent alloreactivity against mismatched primary AML blasts and combination with an CD33/IL-15/CD16 TriKE overcomes resistant blast subtypes

**Figure 5:**
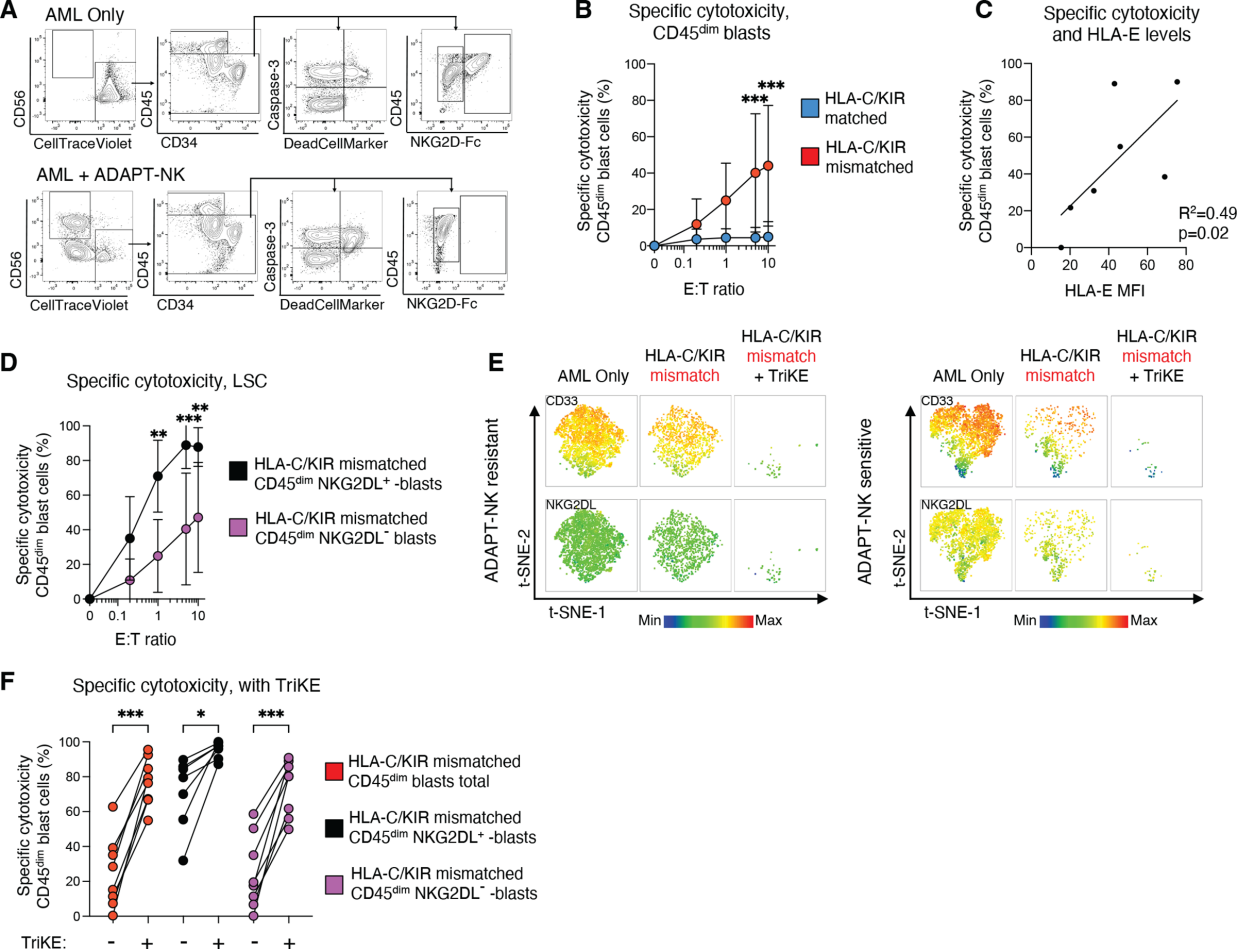
ADAPT-NK cells show potent and specific alloreactivity against mismatched primary AML blasts and combination with CD33/IL-15/CD16 TriKE overcomes resistant blast subtypes. (A) Gating strategy for phenotyping of primary AML blast cells (CD45^dim^) by flow cytometry, as well as specific target cell killing calculated by the change in Dead Cell Marker^+^ Caspase-3^+^ target cells in co-cultures with ADAPT-NK cells. (B) Specific target cell cytotoxicity of AML blasts in HLA-C/KIR -matched and -mismatched conditions at different E:T ratios. (C) Spearman correlation between the mean specific cytotoxicity against each blast sample at 5:1 E:T ratio and the HLA-E expression on the corresponding CD45^dim^ AML blasts. (D) Specific target cell killing of leukemic stem cells (LSC) (NKG2DL^-^) and non-LSCs (NKG2DL^+^) in HLA-C/KIR mismatched conditions at multiple E:T ratios. (E) t-SNE plot of ADAPT-NK sensitive and resistant AML samples co-cultured at 1:1 with HLA-C/KIR mismatched ADAPT-NK cells with and without the addition of CD33/IL-15/CD16 TriKE (TriKE). Heatmap overlays expression of CD33 (top row) and NKG2DL (bottom row). (F) Mean specific cytotoxicity of different blast subsets at 1:1 E:T ratio with or without the addition of TriKE. (A–F) Primary AML samples n=7 and ADAPT-NK products n=8 (overall 14 HLA-C/KIR-matched and 14 HLA-C/KIR-mismatched interactions) in three independent experiments. Statistical differences were tested using a two-way ANOVA followed by Sidak’s multiple comparison correction (B, D), using Spearman’s correlation (C) or using one-way ANOVA followed by Sidak’s multiple comparison correction (F). *p<0.05, **p<0.01, ***p<0.001. AML, acute myeloid leukemia; ANOVA, analysis of variance; E:T, effector to target; KIR, killer immunoglobulin-like receptor; NK, natural killer; t-SNE, t-distributed stochastic neighbor embedding.

Thus far, expanded ADAPT-NK cells showed efficient killing of tumor cell lines in vitro and in vivo. We next tested the ability of NKG2C^+^KIR2DL1^+^KIR2DL3^-^ or KIR2DL3^+^KIR2DL1^-^ ADAPT-NK cells to recognize and eliminate primary AML blasts in PBMC samples derived from patients with distinct HLA-C genotypes. Patient characteristics, including cytogenetics and HLA-C haplotypes, is displayed in online supplemental sFigure 4A. Corroborating the importance of NK cell alloreactivity, minimal NK cell cytotoxicity was observed in HLA-C/KIR matched conditions, whereas mismatched CD45^dim^ AML blasts were efficiently killed at different E:T ratios (figure 5A, B and online supplemental sFigure 4B). The killing of the AML blasts by mismatched ADAPT-NK cells was variable, with some patient samples being more effectively killed and others more resistant. Since the blast compartment of the different samples was heterogeneous, we could monitor putative immune selection events and resistant subtypes. No relation was found between specific cytotoxicity by mismatched ADAPT-NK cells and HLA-DR, CD33, CD34, CD38, CD47, CD112, CD117 or CD155 expression on CD45^dim^ blasts (online supplemental sFigure 4C). Detected levels of HLA-E on blasts were overall low but correlated with sensitivity to killing (figure 5C).

Lack of NKG2D ligand expression is associated with leukemic stem cells (LSC) and involved in mediating their immune evasion.^36^ Although NKG2DL^+^ blasts were eliminated to a higher extent, NKG2DL^-^ LSC were still killed, even at low E:T ratios (figure 5D and online supplemental sFigure 4C). To increase killing of more resistant blast subtypes, we combined mismatched ADAPT-NK cells with a CD16/IL-15/CD33 tri-specific engager (TriKE), previously used to target AML blasts.^37^ The expression of CD33 on AML blasts was high and addition of the TriKE resulted in significantly improved killing of AML blasts, even for more resistant samples and NKG2DL^-^ LSC (figure 5E, F and online supplemental sFigure 4C). Thus, ADAPT-NK cells showed high efficacy against primary AML blasts, and due to their strong ADCC capacity, such expanded adaptive NK cells can be combined with immune engagers to overcome partial NK cell resistance of LSC.

## Discussion

NK cell-based immunotherapy can be a safe and effective treatment for subgroups of leukemia patients with relapsed or refractory disease. Emerging therapeutic approaches focus on enhancing NK cell cytotoxicity and persistence in vivo and making the therapy more readily available in off-the-shelf strategies. One constituent of the NK antitumor response is alloreactive cells in administered NK cell products that relate to better clinical outcomes.^2 10 11^ The preferential expression of a single self-KIR on most adaptive NK cells provides an opportunity to boost the alloreactive response through selective expansion of this NK subset. Adaptive NK cells also inherently display high ADCC capacity and enhanced IFN-γ production.^18 21 24 25^ To harness these properties, we developed a single self-KIR^+^NKG2C^+^ adaptive NK cell product termed ADAPT-NK, using cryopreserved third-party donor material in a fully GMP-compliant protocol. Transcriptional signatures and adaptive features were largely preserved in ADAPT-NK cells post-expansion and the cells exhibited three natural functional modalities without genetic editing: Alloreactivity (single KIR), HLA-E targeting (NKG2C^+^NKG2A^-^) and potent ADCC activity (CD16 expression and signaling).

Generation of homogeneous alloreactive NK populations can theoretically be achieved through (1) selection of single-KIR^+^ NK cells and subsequent expansion,^38^ (2) guided differentiation of precursors with or without genetic editing or (3), as shown here, through selective outgrowth from a mixed NK cell population.^14^ The selective expansion of adaptive NK cells from a mixed NK population avoids any need for GMP-grade selection or editing procedures and therefore reduces the complexity of culturing. Robust cell expansions were generated from all donors with large pre-existing NKG2C^+^ adaptive NK cell subsets, recapitulating the NKG2C phenotype with only slight variation in replicate experiments. This included those donors with almost exclusively CD57^+^NKG2C^+^ adaptive NK cells pre-expansion, suggesting that proliferation could be induced in this subset despite the low proliferative capacity of CD57-expressing cells.^39^

Of note, and in contrast to many cytokine-driven expansion protocols, there was no increase in the fraction of cells expressing NKG2A. In fact, NKG2A expression on NKG2C^+^ ADAPT-NK cells was negligible, resembling NK cell preparations generated by CRISPR editing^40^ or through intracellular retention.^41^ The NKG2A expression remained low also during the phase of intense proliferation coinciding with the disappearance of HLA-E expressing feeders around day 7, suggesting an inheritable adaptive state. Absence of NKG2A-mediated inhibition with concomitant homogeneous expression of NKG2C on ADAPT-NK cells allowed for efficient reactivity toward HLA-E-expressing targets. Hence, in addition to KIR-driven alloreactivity, the switched HLA-E recognition enables targeting of tumors with high HLA-E levels where NKG2A otherwise provides an inhibitory check point.^42^

Adaptive NK cells have a distinct molecular signature including alterations in signaling molecules and surface receptors.^18 22 23^ Transcriptional analysis revealed that although *FCER1G* and *IL12RB* were upregulated the adaptive imprint was largely intact post ADAPT-NK cell expansion. Notably, CD16 and co-stimulatory CD2 expression, critical for the superior ability of adaptive NK cells to respond to antibody-coated target cells,^33^ remained high enabling potent ADCC responses with rituximab and the CD16-IL-15-CD33 TriKE.

Lack of inhibitory input from tumor targets led to ADAPT-NK cell attack in HLA-C/KIR mismatched settings whereas tolerance to self was maintained in matched settings with high HLA-C expression. In addition to NKG2C, the cells displayed high levels of NKG2D, upregulated NKp30 and maintained DNAM-1 levels. They also displayed high levels of effector enzymes such as Granzyme B and perforin, above those of conventional NK cells. Overall, the ADAPT-NK cells showed upregulation of receptors and enrichment of genes related to activation, differentiation, proliferation, cytokine signaling, and regulation of apoptosis and metabolism. Although ADAPT-NK cells expressed surface markers classically associated with T cell exhaustion such as TIM-3, LAG-1 and TIGIT, the transcriptional overlap to exhausted CAR T cells was minimal and did not include the signature marker TOX.^43 44^ Exhaustion in the NK cell compartment is still not well defined. The role of PD-1 on NK cells is controversial and TIGIT and TIM-3 may correlate both with activation/acute stimulation and more exhausted states.^45^ Most importantly, the ADAPT-NK cells showed potent proliferation and cytotoxicity, also in longer-term in vitro assays and displayed serial killing capacity, demonstrating that they were not functionally exhausted.

AML is a heterogeneous disease with varying blast presentations and subtypes. ADAPT-NK cells were highly efficient against an AML cell line in vitro and in vivo as well as against primary AML blasts. Following a single dose of KIR-L mismatched ADAPT-NK cells, tumor burden was significantly reduced in the HL-60 AML mouse model with superior early limitation of tumor growth. Although this in vivo model is extensively used to assess NK cell function,^37 46^ a weakness of the model is the aggressive nature of the tumor with exponential growth as soon as the tumor escapes initial control, which precludes comprehensive survival analyses.

Against primary patient blasts, efficacy of mismatched ADAPT-NK killing was correlated to HLA-E expression on CD45^dim^ blasts as well as NKG2D-L expression and varied greatly among samples. Previous studies have identified the NKG2DL^-^ blasts as particularly NK resistant and able to give rise to new blast populations acting as LSCs.^36^ Therefore, it was particularly important to observe the killing of NKG2DL^-^ blasts even without stimulatory NKG2D input, and an enhanced effect with the TriKE immune engager. This was evident also for blasts carrying poor-prognosis mutations such as FLT3-ITD.^47^ More extensive studies are needed to elucidate resistant and susceptible AML subtypes, also considering the genetic makeup of the AML clone(s), to enable patient stratification in a precision immunotherapy pipeline.

Beneficial features of adaptive NK cells are currently under development from third-party donor cells and engineered iPSC NK cells, mainly focused on NKG2C, FcεRIγ, and CD38 negativity in combination with daratumumab for multiple myeloma.^48 49^ Like the ‘g-NK cells’,^48^ ADAPT-NK cells circumvent the need for genetic engineering while relying on pre-selection of donors harboring adaptive NK subpopulations. Our KIR-centered strategy meant sourcing donors with adaptive NK cells educated by self-KIR2DL1 (HLA-C2/C2 or HLA-C1/C2) or -KIR2DL3 (HLA-C1/C1 or HLA-C1/C2), intended for treating patients with homozygous HLA-C1 or -C2 haplotypes. In preparation for a phase I/II clinical trial for refractory leukemias, we have established a GMP-grade donor cell bank with cryopreserved CD3/CD19 depleted apheresis products from so far nine superdonors (recruitment ongoing), sufficient to manufacture 350 ADAPT-NK doses in an off-the-shelf setting. Although a complete KIR-L mismatch would be the most appealing approach, there is a risk for off-target cytotoxic effects, necessitating precautions in initial dosing regimens. Another important point to consider is the risk for downtuning (or disarming) of NK cell function following adoptive transfer to an HLA disparate recipient, as has been shown in MHC-deficient mice.^50^

In summary, by expanding adaptive single-self-KIR^+^ NKG2C^+^ NK cells in a fully scalable GMP-compliant protocol our study provides the basis to explore alloreactive NK cell therapy. We see potential implications for combination therapies where the ADAPT-NK cell therapy platform is combined with immune engagers or engineering with CARs. If successful, clinical trials based on the ADAPT-NK cells may be able to answer fundamental questions regarding the importance of alloreactivity for GvL effects in hematological malignancies.

10.1136/jitc-2022-005577.supp2 Supplementary data

## Data Availability

Data are available in a public, open access repository. RNA Sequencing data is available at EMBL-EBI European Nucleotide Archive, EGAX. The count matrices have been deposited in the ArrayExpress database under accession number E-MTAB-12228. The raw sequencing data have been deposited to EGA under accession number EGAS00001006614.

## References

[1] Miller JS , Soignier Y , Panoskaltsis-Mortari A , et al . Successful adoptive transfer and in vivo expansion of human haploidentical NK cells in patients with cancer. Blood 2005;105:3051–7. 10.1182/blood-2004-07-2974 15632206

[2] Curti A , Ruggeri L , D'Addio A , et al . Successful transfer of alloreactive haploidentical Kir ligand-mismatched natural killer cells after infusion in elderly high risk acute myeloid leukemia patients. Blood 2011;118:3273–9. 10.1182/blood-2011-01-329508 21791425

[3] Romee R , Rosario M , Berrien-Elliott MM , et al . Cytokine-Induced memory-like natural killer cells exhibit enhanced responses against myeloid leukemia. Sci Transl Med 2016;8:ra123. 10.1126/scitranslmed.aaf2341 PMC5436500 27655849

[4] Ciurea SO , Schafer JR , Bassett R , et al . Phase 1 clinical trial using mbIL21 ex vivo-expanded donor-derived NK cells after haploidentical transplantation. Blood 2017;130:1857–68. 10.1182/blood-2017-05-785659 28835441 PMC5649552

[5] Björklund AT , Carlsten M , Sohlberg E , et al . Complete remission with reduction of high-risk clones following haploidentical NK-cell therapy against MDS and AML. Clin Cancer Res 2018;24:1834–44. 10.1158/1078-0432.CCR-17-3196 29444931

[6] Myers JA , Miller JS . Exploring the NK cell platform for cancer immunotherapy. Nat Rev Clin Oncol 2021;18:85–100. 10.1038/s41571-020-0426-7 32934330 PMC8316981

[7] Horowitz A , Strauss-Albee DM , Leipold M , et al . Genetic and environmental determinants of human NK cell diversity revealed by mass cytometry. Sci Transl Med 2013;5:ra145. 10.1126/scitranslmed.3006702 PMC3918221 24154599

[8] Ljunggren HG , Kärre K . In search of the 'missing self': MHC molecules and NK cell recognition. Immunol Today 1990;11:237–44. 10.1016/0167-5699(90)90097-S 2201309

[9] Kärre K . Immunology. A perfect mismatch. Science 2002;295:2029–31. 10.1126/science.1070538 11896262

[10] Ruggeri L , Capanni M , Urbani E , et al . Effectiveness of donor natural killer cell alloreactivity in mismatched hematopoietic transplants. Science 2002;295:2097–100. 10.1126/science.1068440 11896281

[11] Ruggeri L , Mancusi A , Burchielli E , et al . Natural killer cell alloreactivity in allogeneic hematopoietic transplantation. Curr Opin Oncol 2007;19:142–7. 10.1097/CCO.0b013e3280148a1a 17272987

[12] Fauriat C , Andersson S , Björklund AT , et al . Estimation of the size of the alloreactive NK cell repertoire: studies in individuals homozygous for the group A Kir haplotype. J Immunol 2008;181:6010–9. 10.4049/jimmunol.181.9.6010 18941190

[13] Yawata M , Yawata N , Draghi M , et al . MHC class I-specific inhibitory receptors and their ligands structure diverse human NK-cell repertoires toward a balance of missing self-response. Blood 2008;112:2369–80. 10.1182/blood-2008-03-143727 18583565 PMC2532809

[14] Liu LL , Pfefferle A , Yi Sheng VO , et al . Harnessing adaptive natural killer cells in cancer immunotherapy. Mol Oncol 2015;9:1904–17. 10.1016/j.molonc.2015.10.001 26604011 PMC5528731

[15] Gumá M , Angulo A , Vilches C , et al . Imprint of human cytomegalovirus infection on the NK cell receptor repertoire. Blood 2004;104:3664–71. 10.1182/blood-2004-05-2058 15304389

[16] Lopez-Vergès S , Milush JM , Schwartz BS , et al . Expansion of a unique CD57⁺NKG2Chi natural killer cell subset during acute human cytomegalovirus infection. Proc Natl Acad Sci U S A 2011;108:14725–32. 10.1073/pnas.1110900108 21825173 PMC3169160

[17] Gumá M , Budt M , Sáez A , et al . Expansion of CD94/NKG2C+ NK cells in response to human cytomegalovirus-infected fibroblasts. Blood 2006;107:3624–31. 10.1182/blood-2005-09-3682 16384928

[18] Béziat V , Liu LL , Malmberg J-A , et al . NK cell responses to cytomegalovirus infection lead to stable imprints in the human Kir repertoire and involve activating KIRs. Blood 2013;121:2678–88. 10.1182/blood-2012-10-459545 23325834 PMC3617633

[19] Rölle A , Pollmann J , Ewen E-M , et al . IL-12-producing monocytes and HLA-E control HCMV-driven NKG2C+ NK cell expansion. J Clin Invest 2014;124:5305–16. 10.1172/JCI77440 25384219 PMC4348979

[20] Hammer Q , Rückert T , Borst EM , et al . Peptide-specific recognition of human cytomegalovirus strains controls adaptive natural killer cells. Nat Immunol 2018;19:453–63. 10.1038/s41590-018-0082-6 29632329

[21] Luetke-Eversloh M , Hammer Q , Durek P , et al . Human cytomegalovirus drives epigenetic imprinting of the IFNG locus in NKG2Chi natural killer cells. PLoS Pathog 2014;10:e1004441. 10.1371/journal.ppat.1004441 25329659 PMC4199780

[22] Schlums H , Cichocki F , Tesi B , et al . Cytomegalovirus infection drives adaptive epigenetic diversification of NK cells with altered signaling and effector function. Immunity 2015;42:443–56. 10.1016/j.immuni.2015.02.008 25786176 PMC4612277

[23] Holmes TD , Pandey RV , Helm EY , et al . The transcription factor Bcl11b promotes both canonical and adaptive NK cell differentiation. Sci Immunol 2021;6:eabc9801. 10.1126/sciimmunol.abc9801 33712472 PMC8274449

[24] Zhang T , Scott JM , Hwang I , et al . Cutting edge: antibody-dependent memory-like NK cells distinguished by FcRγ deficiency. J Immunol 2013;190:1402–6. 10.4049/jimmunol.1203034 23345329 PMC3623944

[25] Luetke-Eversloh M , Killig M , Romagnani C . Signatures of human NK cell development and terminal differentiation. Front Immunol 2013;4:499. 10.3389/fimmu.2013.00499 24416035 PMC3874559

[26] Cichocki F , Cooley S , Davis Z , et al . CD56dimCD57+NKG2C+ NK cell expansion is associated with reduced leukemia relapse after reduced intensity hCT. Leukemia 2016;30:456–63. 10.1038/leu.2015.260 26416461 PMC4740203

[27] Takenaka K , Nishida T , Asano-Mori Y , et al . Cytomegalovirus reactivation after allogeneic hematopoietic stem cell transplantation is associated with a reduced risk of relapse in patients with acute myeloid leukemia who survived to day 100 after transplantation: the Japan Society for hematopoietic cell transplantation Transplantation-related complication Working group. Biol Blood Marrow Transplant 2015;21:2008–16. 10.1016/j.bbmt.2015.07.019 26211985

[28] Green ML , Leisenring WM , Xie H , et al . Cmv reactivation after allogeneic hCT and relapse risk: evidence for early protection in acute myeloid leukemia. Blood 2013;122:1316–24. 10.1182/blood-2013-02-487074 23744585 PMC3744995

[29] Elmaagacli AH , Steckel NK , Koldehoff M , et al . Early human cytomegalovirus replication after transplantation is associated with a decreased relapse risk: evidence for a putative virus-versus-leukemia effect in acute myeloid leukemia patients. Blood 2011;118:1402–12. 10.1182/blood-2010-08-304121 21540462

[30] Liu LL , Béziat V , Oei VYS , et al . Ex Vivo Expanded Adaptive NK Cells Effectively Kill Primary Acute Lymphoblastic Leukemia Cells. Cancer Immunol Res 2017;5:654–65. 10.1158/2326-6066.CIR-16-0296 28637877

[31] Basha O , Shpringer R , Argov CM , et al . The DifferentialNet database of differential protein-protein interactions in human tissues. Nucleic Acids Res 2018;46:D522–6. 10.1093/nar/gkx981 29069447 PMC5753382

[32] Guldevall K , Brandt L , Forslund E , et al . Microchip screening platform for single cell assessment of NK cell cytotoxicity. Front Immunol 2016;7:119. 10.3389/fimmu.2016.00119 27092139 PMC4820656

[33] Liu LL , Landskron J , Ask EH , et al . Critical role of CD2 co-stimulation in adaptive natural killer cell responses revealed in NKG2C-Deficient humans. Cell Rep 2016;15:1088–99. 10.1016/j.celrep.2016.04.005 27117418 PMC4858565

[34] Collins PL , Cella M , Porter SI , et al . Gene regulatory programs conferring phenotypic identities to human NK cells. Cell 2019;176:348–60. 10.1016/j.cell.2018.11.045 30595449 PMC6329660

[35] Blank CU , Haining WN , Held W , et al . Defining 'T cell exhaustion'. Nat Rev Immunol 2019;19:665–74. 10.1038/s41577-019-0221-9 31570879 PMC7286441

[36] Paczulla AM , Rothfelder K , Raffel S , et al . Absence of NKG2D ligands defines leukaemia stem cells and mediates their immune evasion. Nature 2019;572:254–9. 10.1038/s41586-019-1410-1 31316209 PMC6934414

[37] Vallera DA , Felices M , McElmurry R , et al . Il15 trispecific killer Engagers (TriKE) make natural killer cells specific to CD33+ targets while also inducing persistence, in vivo expansion, and enhanced function. Clin Cancer Res 2016;22:3440–50. 10.1158/1078-0432.CCR-15-2710 26847056 PMC4947440

[38] Siegler U , Meyer-Monard S , Jörger S , et al . Good manufacturing practice-compliant cell sorting and large-scale expansion of single KIR-positive alloreactive human natural killer cells for multiple infusions to leukemia patients. Cytotherapy 2010;12:750–63. 10.3109/14653241003786155 20491532

[39] Björkström NK , Riese P , Heuts F , et al . Expression patterns of NKG2A, Kir, and CD57 define a process of CD56dim NK-cell differentiation uncoupled from NK-cell education. Blood 2010;116:3853–64. 10.1182/blood-2010-04-281675 20696944

[40] Berrien-Elliott MM , Cashen AF , Cubitt CC , et al . Multidimensional analyses of donor Memory-Like NK cells reveal new associations with response after adoptive immunotherapy for leukemia. Cancer Discov 2020;10:1854–71. 10.1158/2159-8290.CD-20-0312 32826231 PMC7710923

[41] Kamiya T , Seow SV , Wong D , et al . Blocking expression of inhibitory receptor NKG2A overcomes tumor resistance to NK cells. J Clin Invest 2019;129:2094–106. 10.1172/JCI123955 30860984 PMC6486333

[42] van Hall T , André P , Horowitz A , et al . Monalizumab: inhibiting the novel immune checkpoint NKG2A. J Immunother Cancer 2019;7:263. 10.1186/s40425-019-0761-3 31623687 PMC6798508

[43] Alfei F , Kanev K , Hofmann M , et al . Tox reinforces the phenotype and longevity of exhausted T cells in chronic viral infection. Nature 2019;571:265–9. 10.1038/s41586-019-1326-9 31207605

[44] Marotel M , Villard M , Drouillard A , et al . Peripheral natural killer cells in chronic hepatitis B patients display multiple molecular features of T cell exhaustion. Elife 2021;10:e60095. 10.7554/eLife.60095 33507150 PMC7870135

[45] Judge SJ , Murphy WJ , Canter RJ . Characterizing the dysfunctional NK cell: assessing the clinical relevance of exhaustion, anergy, and senescence. Front Cell Infect Microbiol 2020;10:49. 10.3389/fcimb.2020.00049 32117816 PMC7031155

[46] Felices M , Lenvik TR , Kodal B , et al . Potent cytolytic activity and specific IL15 delivery in a second-generation trispecific killer Engager. Cancer Immunol Res 2020;8:1139–49. 10.1158/2326-6066.CIR-19-0837 32661096 PMC7484162

[47] Daver N , Schlenk RF , Russell NH , et al . Targeting FLT3 mutations in AML: review of current knowledge and evidence. Leukemia 2019;33:299–312. 10.1038/s41375-018-0357-9 30651634 PMC6365380

[48] Bigley AB , Spade S , Agha NH , et al . FcεRIγ-negative NK cells persist in vivo and enhance efficacy of therapeutic monoclonal antibodies in multiple myeloma. Blood Adv 2021;5:3021–31. 10.1182/bloodadvances.2020002440 34357379 PMC8361460

[49] Woan KV , Kim H , Bjordahl R , et al . Harnessing features of adaptive NK cells to generate iPSC-derived NK cells for enhanced immunotherapy. Cell Stem Cell 2021;28:2062–75. 10.1016/j.stem.2021.08.013 34525347 PMC8642276

[50] Joncker NT , Shifrin N , Delebecque F , et al . Mature natural killer cells reset their responsiveness when exposed to an altered MHC environment. J Exp Med 2010;207:2065–72. 10.1084/jem.20100570 20819928 PMC2947079

