# Correction: Adaptive single-KIR^+^NKG2C^+^ NK cells expanded from select superdonors show potent missing-self reactivity and efficiently control HLA-mismatched acute myeloid leukemia

**DOI:** 10.1136/jitc-2022-005577corr1

**Published:** 2023-01-24

Haroun-Izquierdo A, Vincenti M, Netskar H, *et al*. Adaptive single-KIR+NKG2C+ NK cells expanded from select superdonors show potent missing-self reactivity and efficiently control HLA-mismatched acute myeloid leukemia. *J Immunother Cancer* 2022;10:e005577. doi: 10.1136/jitc-2022-005577

This article has been updated since it was first published online. The paragraph under the heading ‘ADAPT-NK cell expansion protocol’ has been updated to include the sentence ‘10% human ab serum (TCS Biosciences or Access Biologicals), 2mM L-glutamine (Cytiva-FisherScientific)’.

