# Supplementary material for: Adaptive single-KIR+NKG2C+ NK cells expanded from select superdonors show potent missing-self reactivity and efficiently control HLA-mismatched acute myeloid leukemia
Source: J Immunother Cancer. 2022 Nov 1;10(11):e005577. doi: 10.1136/jitc-2022-005577 (PMC9628692; doi:10.1136/jitc-2022-005577)
Supplement: Supplementary data [file jitc-2022-005577supp001.pdf]

Haroun-Izquierdo et al, Supplemental Material

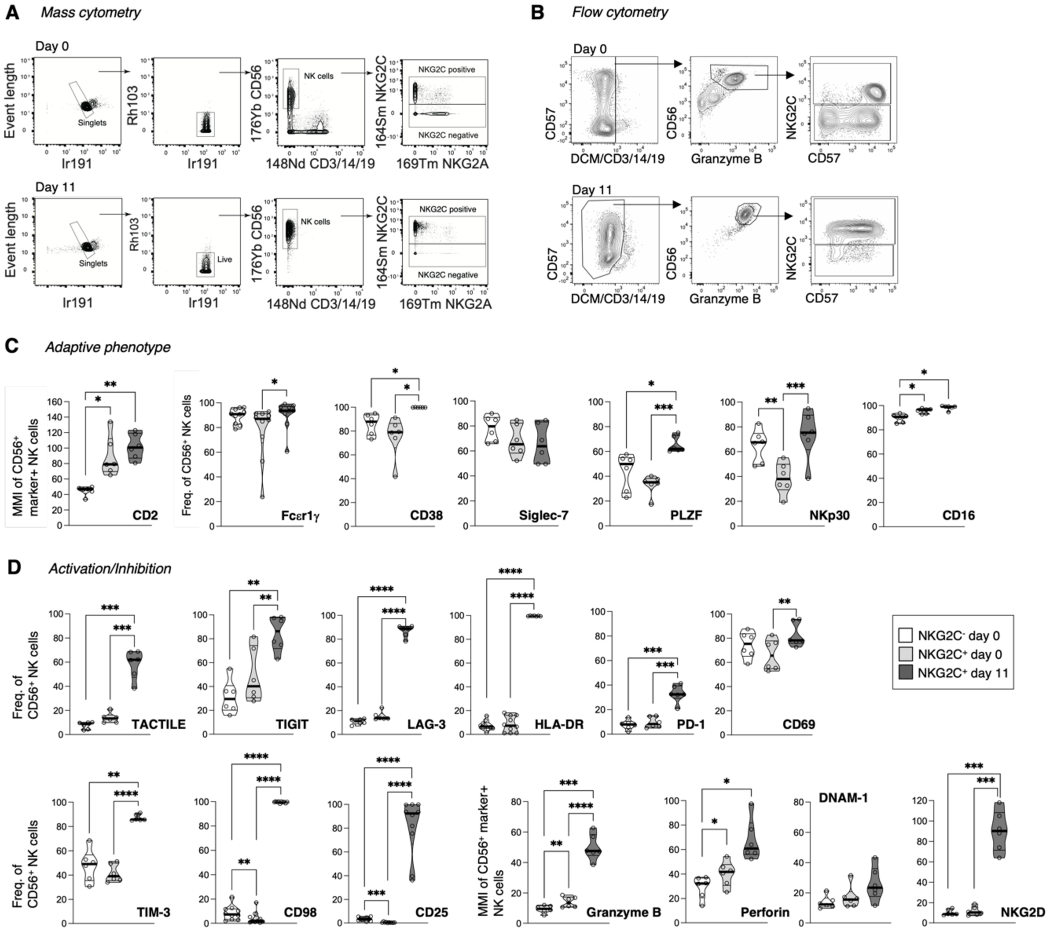

Supplemental Figure 1. Extended phenotyping by flow- and mass cytometry of ADAPT-NK cells. **A)** Gating scheme for mass cytometry analysis of NKG2C^{+} and NKG2C^{-} CD56^{+} NK cells. **B)** Gating scheme for flow cytometry analysis of NKG2C^{+} and NKG2C^{-} CD56^{+} NK cells, gated upstream on fsc-h/a and ssc-h/a single cells. **C-D)** Summary data that are the basis of heatmaps in Fig. 2B, frequencies of marker expression or mean metal intensity (MMI). Data for Fcεr1γ, HLA-DR, CD98 and CD25 derive from flow cytometry data. p-value <0.05 * <0.01 ** <0.001 *** <0.0001 **** in one-way ANOVA with Tukey's correction (C-D). For flow- and mass cytometry n=4-7.

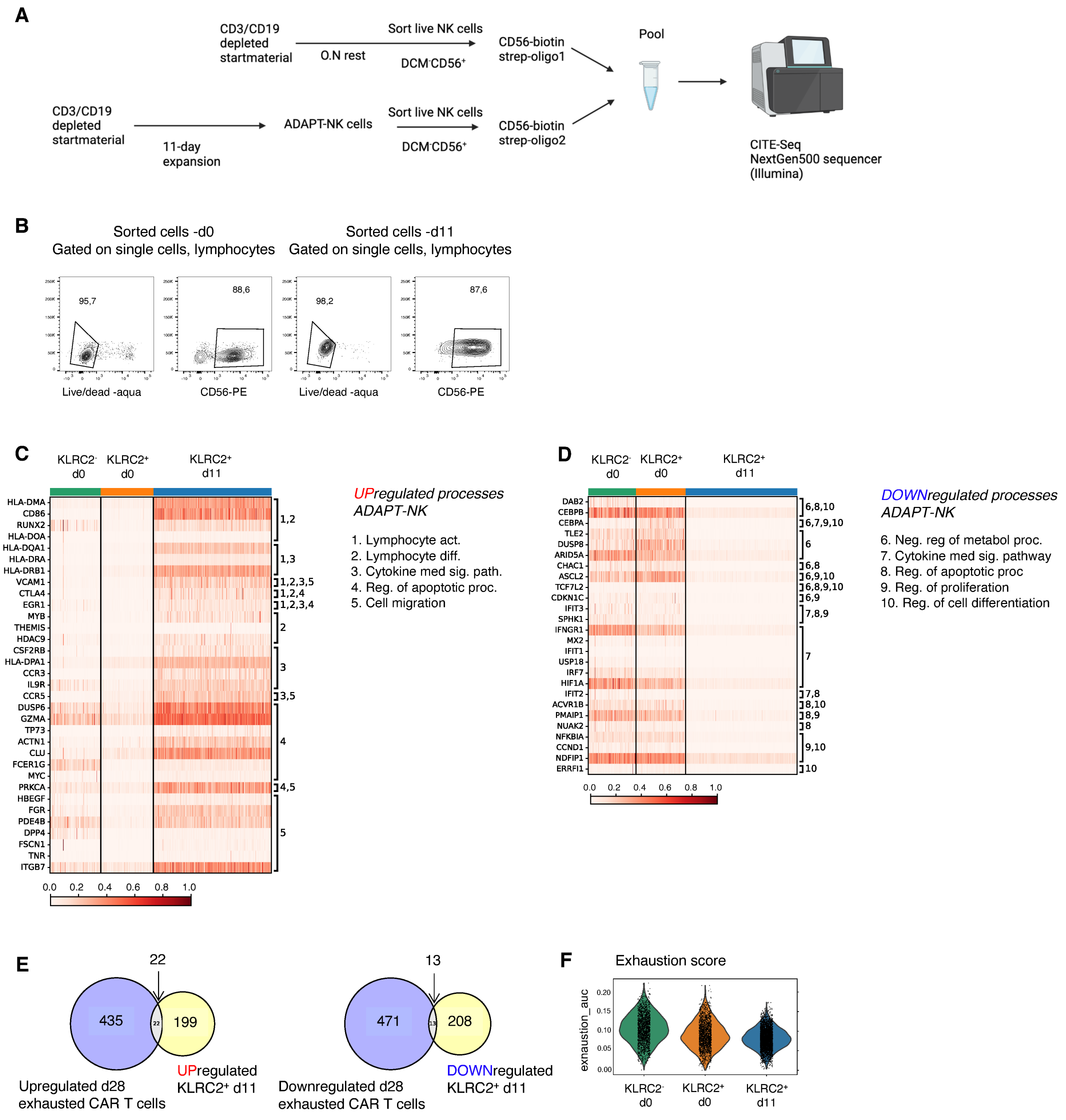

Supplemental Figure 2. ADAPT-NK cells show a retained adaptive transcriptional signature with activation of effector programs. A) CITE-Seq workflow **B)** Sort gates for day 0 and day 11 NK cells **C-D)** Heatmaps showing the expression of the upregulated **(C)** and downregulated (**D**) genes in ADAPT-NK in the CITE-seq data sorted by the identified clusters and showing the GO (BP) processes associated with these genes **E)** Venn diagram showing the overlap between the up- and down-regulated genes in the KLRC2^{+} day 11 cluster and up- and down-regulated genes in exhausted CAR T cells (Good et al., [1]). **F)** Exhaustion score computed using AUCell showing the activity of this signature in the clusters. CITE-Seq n=1.

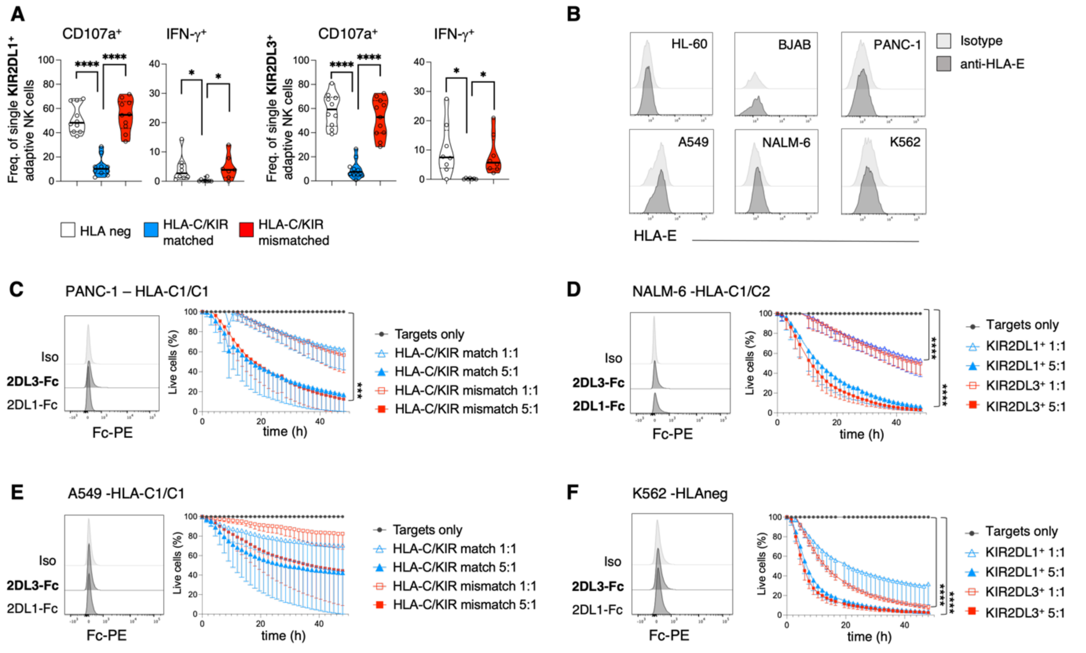

Supplemental Figure 3. ADAPT-NK cells are highly functional with predictable alloreactivity. Functional analysis of NK cells by flow cytometry in terms of degranulation (CD107a) and IFN-γ production. **A)** Responses of NKG2C$^+$ KIR2DL1$^+$ KIR2DL3$^-$ KIR2DL2$^-$ NKG2A$^-$ or NKG2C$^+$ KIR2DL1$^-$ KIR2DL3$^+$ KIR2DL2$^-$ NKG2A$^-$ ADAPT-NK cells to K562 with single chain β2m HLA-C1 or HLA-C2 dimers, in the HLA-C/KIR matched and mismatched setting. n=5-11, in 3 independent experiments with KIR2DL1$^+$ and KIR2DL3$^+$ subsets analyzed in all donors. **B)** Determination of HLA-E expression on tumor target lines. **C-F)** Determination of killing ability of ADAPT-NK cells over 48 h against tumor cell lines of various HLA-C genotypes and at different effector to target ratios (1:1 and 5:1) in the IncuCyte platform. In bold the expected KIR-Fc binding. n=4-9 donors, in at least two independent experiments for each target cell line. Data is displayed as mean (± SD) and significance is given between HLA-C/KIR mismatched and targets only. p-value <0.05 * <0.01 ** <0.001 *** <0.0001 **** in one-way ANOVA followed by Sidak's multiple comparison correction (C-F).

A

Patient sample	Diagnosis	Mutation	HLA-C
AML#1	AML	FLT3-ITD	C1/C1
AML#2	AML	no data	C1/C1
AML#3	AML	no data	C1/C1
AML#4	AML	FLT3-ITD	C2/C2
AML#5	AML	CEBP	C2/C2
AML#6	AML	FLT3-ITD	C1/C1
AML#7	AML	no data	C1/C1
AML#8	AML	NPMI1	C2/C2

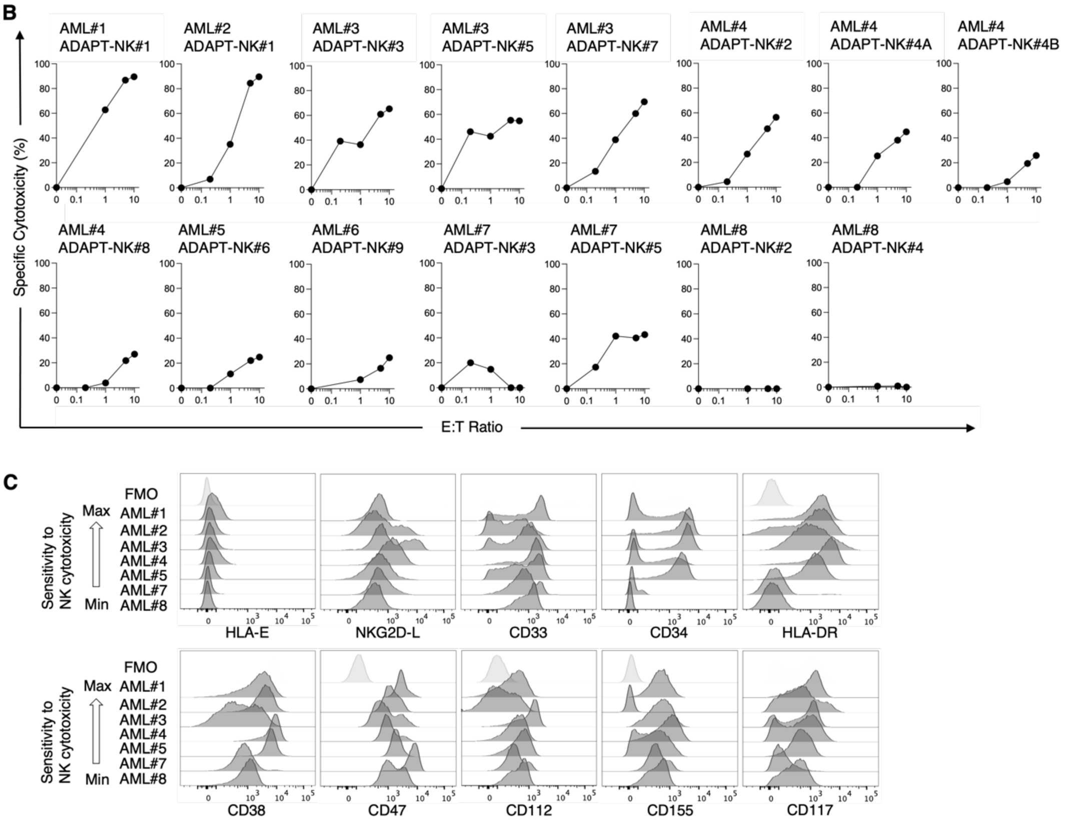

Supplemental Figure 4. Cytotoxicity of ADAPT-NK cells against primary AML blasts. A) AML patient cytogenetics and HLA-C haplotypes. **B)** Cytotoxicity against 8 primary AML blasts by 9 different HLA-C/KIR-mismatched ADAPT-NK products in 3 independent experiments. Some AML samples were co-cultured with multiple NK products (up to 3) in separate experiments. A and B notation on ADAPT-NK#4 denotes a repeat combination in 2 independent experiments. **C)** Surface marker expression of indicated proteins evaluated by flow cytometry on 7 CD45^{dim} AML blasts and ordered based on decreased sensitivity to ADAPT-NK cell cytotoxicity, FMO – fluorescence minus one where indicated.

Supplemental table 1. mAbs used for Flow cytometry

Fluorochrome	Antigen	Clone	Vendor
**APC**	Anti-Biotin	REA746	Miltenyi
**BV785**	CD3	UCHT1	BD Biosciences
**PE-Cy5**	CD3	UHCT1	Beckman Coulter
**ECD**	CD3	UCHT1	Beckman Coulter
**BV421**	CD3	SK7	Biolegend
**APC**	CD3	HIT3a	BD Biosciences
**V500**	CD14	MφP9	BD Biosciences
**BV570**	CD14	M5E2	Biolegend
**ECD**	CD14	RMO52	Beckman Coulter
**FITC**	CD14	M5E2	BD Biosciences
**BUV395**	CD16	3G8	BD Biosciences
**ECD**	CD16	3G8	Beckman Coulter
**BV785**	CD16	3G8	Biolegend
**V500**	CD19	HIB19	BD Biosciences
**BV570**	CD19	HIB19	Biolegend
**ECD**	CD19	J3-119	Beckman Coulter
**BV510**	CD19	SJ25C1	BD Biosciences
**BV421**	CD25	2A3	BD Biosciences
**PE-Vio770**	CD33	REA775	Miltenyi
**BUV395**	CD34	581	BD Biosciences
**BV711**	CD38	HIT2	Biolegend
**BV650**	CD45	HI30	Biolegend
**PerCP-Cy5.5**	CD45	H130	BD Biosciences
**BV421**	CD47	B6H12	BD Biosciences
**BUV737**	CD56	NCAM16.2	BD Biosciences
**ECD**	CD56	NHK-1	Beckman Coulter
**PE-Cy7**	CD56	NCAM16.2	BD Biosciences
**Pacific Blue**	CD57	HCD57	Biolegend
**BV650**	CD98	UM7F8	BD Biosciences
**BUV395**	CD107a	H4A3	BD Biosciences
**APC**	CD112	TX31	Biolegend
**PE-Cy5.5**	CD117	104D2D1	Beckman Coulter
**PE**	CD155	SKII.4	Biolegend
**PE**	EPCAM	9C4 (#271)	Biolegend
**FITC**	FcεRIg	polyclonal	Merck-Millipore
**BV785**	HLA-DR	L243	Biolegend
**APC**	HLA-E	3D12	Biolegend

**PE**	HLA-E	3D12	Biolegend
**PE-Cy7**	HLA-E	3D12	Biolegend
**AF700**	IFN-γ	B27	BD Biosciences
**BV785**	IFN-γ	4S.B3	Biolegend
**APC**	KIR2DL1	REA284	Miltenyi
**PE-Cy7**	KIR2DL1/S1	EB6B	Beckman Coulter
**Biotin**	KIR2DL3	REA147	Miltenyi
**PE-Cy5.5**	KIR2DL3/L2/S2	GL183	Beckman Coulter
**AF700**	KIR3DL1	DX9	Biolegend
**APC-Fire750**	KIR3DL1	DX9	Biolegend
**Vio-Bright-FITC**	NKG2A	REA110	Miltenyi
**PE**	NKG2C	FAB138P	R&D Systems
**APC**	NKG2C	REA205	Miltenyi
**PE**	NKG2C	REA205	Miltenyi
**BV711**	Streptavidin		BD Biosciences
**PE**	TIGIT	MBSA43	eBiosciences – Thermo Fisher Scientific
**BV650**	TNF	MAb11	Biolegend
**Aqua**	LIVE/DEAD Fixable Aqua Dead Cell Stain Kit		Thermo Fisher Scientific
**Near-IR**	LIVE/DEAD Fixable Near-IR Dead Cell Stain Kit		Thermo Fisher Scientific
**eFluor780**	eBiosciences Fixable Viability Dye		Thermo Fisher Scientific
	CellTrace™ Violet Cell Proliferation Kit		Thermo Fisher Scientific
**FITC**	Cleaved Caspase-3 Staining Kit (FITC)		Abcam
	Recombinant Human KIR2DL1/CD158a Fc Chimera Protein	#1844-KR-050	R&D Systems
	Recombinant Human KIR2DL3/CD158b2 Fc Chimera Protein	#2014-KR-050	R&D Systems
	Recombinant Human NKG2D Fc Chimera Protein		R&D Systems
**PE**	Goat anti-Human IgG Fc Secondary Antibody	polyclonal	Thermo Fisher Scientific

Supplemental table 2. mAbs used for Mass cytometry

Mass	Antigen	Clone	Vendor
**89Y**	CD45	HI30	Fluidigm
**141Pr**	CX3CR1	REA385	Miltenyi
**142Nd**	CD57	HCD57	Fluidigm
**143Nd**	CD2	TS1/8	Biolegend
**144Nd**	CD38	REA572	Miltenyi
**145Nd**	CXCR3	REA232	Miltenyi
**146Nd**	CCR2	REA538	Miltenyi
**147Sm**	CD96	NK92.39	Biolegend
**148Nd**	CD3	OKT3	Biolegend
**148Nd**	CD14	RMO52	Fluidigm
**148Nd**	CD19	HIB19	Biolegend
**149Sm**	FasL	NOK-1	Biolegend
**150Nd**	LAG3	11C3C65	Fluidigm
**151Eu**	CXCR4	REA649	Miltenyi
**152Sm**	Siglec-7	194211	Fluidigm
**153Eu**	TIM-3	F38-2E2	Fluidigm
**154Sm**	NKG2C	REA205	Miltenyi
**155Gd**	CXCR1	REA958	Miltenyi
**156Gd**	KIR2DL1/S1	11PB6	Miltenyi
**158Gd**	KIR2DL1	REA284	Miltenyi
**159Tb**	GITR	108-17	Biolegend
**160Gd**	GPR56	CG4	Biolegend
**161Dy**	PLZF	17.10,.17	Biolegend
**162Dy**	CD69	FN50	Fluidigm
**163Dy**	KIR2DL2/S2/L3	GL183	Beckman Coulter
**164Dy**	TIGIT	MBSA43	Thermo Fisher Scientific
**165Ho**	CCR5	REA245	Miltenyi
**166Er**	NKG2D	ON72	Fluidigm
**167Er**	CCR7	REA108	Miltenyi
**168Er**	NKp30	P30-15	Miltenyi
**169Tm**	NKG2A	Z199	Fluidigm
**170Er**	CXCR2	REA208	Miltenyi
**171Yb**	DNAM-1	DX11	Fluidigm
**172Yb**	KIR3DL1	DX9	R&D
**173Yb**	Granzyme B	GB11	Fluidigm
**174Yb**	PD-1	EH12.2H7	Fluidigm
**175Lu**	Perforin	B-D48	Fluidigm
**176Yb**	CD56	NCAM16.2	Fluidigm
**209Bi**	CD16	3G8	Fluidigm

Supplemental table 3. GO terms for upregulated genes in Steiner Forest PPI network

Pathway	Total	Expected	Hits	FDR
Cell activation	960	12.6	50	9.35e-15
Leukocyte activation	707	9.3	39	3.9e-12
Lymphocyte activation	601	7.91	36	3.9e-12
Regulation of immune response	727	9.57	39	7.11e-12
Immune response	1430	18.8	55	1.48e-11
Leukocyte differentiation	404	5.32	28	7.03e-11
Regulation of immune system process	1190	15.7	48	1.37e-10
Positive regulation of lymphocyte activation	250	3.29	22	1.83e-10
Hemopoiesis	640	8.42	34	2.15e-10
T cell activation	432	5.68	28	2.15e-10
Lymphocyte differentiation	260	3.42	22	2.92e-10
B cell activation	194	2.55	19	6.62e-10
Hematopoietic or lymphoid organ development	679	8.93	34	8.41e-10
Immune system development	722	9.5	35	9.24e-10
Immune system process	2720	35.8	75	1.09e-09
Cytokine-mediated signaling pathway	374	4.92	25	1.15e-09
Positive regulation of immune system process	739	9.72	34	6.35e-09
Positive regulation of T cell activation	202	2.66	18	7.67e-09
Regulation of lymphocyte activation	360	4.74	23	1.59e-08
B cell differentiation	82	1.08	12	2.88e-08
Regulation of T cell activation	279	3.67	20	2.98e-08
T cell differentiation	186	2.45	16	1.14e-07
Regulation of response to stimulus	3360	44.2	80	1.78e-07
Positive regulation of response to stimulus	1550	20.4	48	3.52e-07
Response to stress	4150	54.6	91	4.62e-07
Positive regulation of immune response	487	6.41	24	8.01e-07
Response to wounding	1310	17.3	42	1.29e-06
Regulation of body fluid levels	680	8.95	28	2.19e-06
Blood coagulation	564	7.42	24	1.1e-05
Coagulation	568	7.47	24	1.21e-05
Hemostasis	570	7.5	24	1.24e-05
Response to radiation	345	4.54	18	1.79e-05
Defense response	1510	19.9	43	1.79e-05
Interaction with host	426	5.61	20	2.08e-05
Regulation of immune effector process	249	3.28	15	2.44e-05
Production of molecular mediator of immune response	130	1.71	11	2.53e-05
Regulation of apoptotic process	1540	20.2	43	2.53e-05
Activation of immune response	399	5.25	19	2.88e-05
Regulation of programmed cell death	1550	20.4	43	3.27e-05
Wound healing	700	9.21	26	3.33e-05
Immune effector process	576	7.58	23	4.12e-05
Response to organic substance	2500	32.9	59	4.18e-05
Innate immune response	638	8.39	24	6.52e-05
Regulation of cell differentiation	1290	17	37	7.99e-05
Positive regulation of cell proliferation	786	10.3	27	7.99e-05
Cell migration	1050	13.9	32	0.000125
Positive regulation of developmental process	817	10.8	27	0.000156
Peptidyl tyrosine phosphorylation	228	3	13	0.000173
Positive regulation of metabolic process	2690	35.4	60	0.000183
Peptidyl tyrosine modification	230	3.03	13	0.000183
Viral reproductive process	597	7.86	22	0.000201
Regulation of cellular component organization	1520	20.1	40	0.000226
Response to ionizing radiation	112	1.47	9	0.000256
Regulation of developmental process	1880	24.7	46	0.000256
Regulation of protein metabolic process	1820	24	45	0.000268
Adaptive immune response	241	3.17	13	0.000268
Inflammatory response	569	7.49	21	0.00028
Regulation of myeloid cell differentiation	145	1.91	10	0.000307
Cell proliferation	1900	25	46	0.000329
Positive regulation of biological process	5500	72.3	100	0.000361
Positive regulation of cellular metabolic process	2530	33.2	56	0.000389
Negative regulation of apoptotic process	679	8.93	23	0.000389
Response to abiotic stimulus	876	11.5	27	0.000395
Adaptive immune response based on somatic recombination of immune receptors built from immunoglobulin superfamily domains	218	2.87	12	4,00E-04
Regulation of protein modification process	1250	16.4	34	0.000422
Negative regulation of programmed cell death	691	9.09	23	0.000481
Negative regulation of signal transduction	790	10.4	25	0.000491

Positive regulation of cell migration	263	3.46	13	0.000542
Cell surface receptor signaling pathway	3340	43.9	68	0.000542
Positive regulation of cellular process	4780	62.9	89	0.000571
Response to light stimulus	229	3.01	12	0.000584
Homeostasis of number of cells	196	2.58	11	0.000641
Exocytosis	309	4.07	14	0.000656
Leukocyte migration	309	4.07	14	0.000656
Interphase of mitotic cell cycle	435	5.72	17	0.000674
Positive regulation of cell differentiation	571	7.51	20	0.000688
Regulation of multicellular organismal process	2480	32.6	54	0.000748
Epidermal growth factor receptor signaling pathway	167	2.2	10	0.000752
Intrinsic apoptotic signaling pathway	135	1.78	9	0.000752
Interphase	443	5.83	17	0.000783
Positive regulation of protein metabolic process	1080	14.3	30	0.000783
Apoptotic process	2130	28	48	0.000877
Positive regulation of transcription, DNA-dependent	1260	16.6	33	0.000901
Regulation of cell proliferation	1430	18.8	36	0.000917
Peptidyl amino acid modification	739	9.72	23	0.00101
Response to UV	112	1.47	8	0.00106
Regulation of cellular protein metabolic process	1560	20.5	38	0.0011
Programmed cell death	2160	28.4	48	0.00111
Negative regulation of transcription from RNA polymerase II promoter	552	7.26	19	0.00111
Positive regulation of RNA metabolic process	1330	17.5	34	0.00111
T cell proliferation	147	1.93	9	0.00123
Myeloid cell differentiation	296	3.89	13	0.0013
Negative regulation of metabolic process	1820	24	42	0.00148
Regulation of transferase activity	768	10.1	23	0.00161
Nucleocytoplasmic transport	388	5.11	15	0.00161
Nuclear transport	392	5.16	15	0.00178
Positive regulation of cytokine biosynthetic process	66	0.868	6	0.00184
Positive regulation of nucleobase-containing compound metabolic process	1490	19.6	36	0.00184
Regulation of mitotic cell cycle	351	4.62	14	0.00184
Regulation of cell cycle	886	11.7	25	0.00198
Regulation of kinase activity	743	9.78	22	0.00246
Regulation of phosphorylation	1070	14	28	0.00266
Regulation of protein kinase activity	698	9.18	21	0.00269
Viral reproduction	803	10.6	23	0.00278
Interleukin_2 production	47	0.618	5	0.00278
Negative regulation of cellular component organization	370	4.87	14	0.00292
Negative regulation of response to stimulus	967	12.7	26	0.00292
Protein complex assembly	861	11.3	24	0.00295
Regulation of peptidyl tyrosine phosphorylation	171	2.25	9	0.00319
Regulation of cytokine production	513	6.75	17	0.00321
Positive regulation of T cell proliferation	75	0.987	6	0.00326
Negative regulation of cellular metabolic process	1660	21.9	38	0.00326
Regulation of cell-cell adhesion	77	1.01	6	0.00368
Regulation of protein phosphorylation	987	13	26	0.00368
Negative regulation of transcription, DNA-dependent	987	13	26	0.00368
Cellular response to stress	1620	21.4	37	0.00398

Supplemental table 4. GO terms for downregulated genes in Steiner Forest PPI network

Pathway	Total	Expected	Hits	FDR
Negative regulation of metabolic process	1820	19.9	55	2.37e-10
Negative regulation of cellular metabolic process	1660	18.2	52	2.37e-10
Negative regulation of signal transduction	790	8.63	34	9.87e-10
Negative regulation of cellular process	4110	44.8	85	2.4e-09
Regulation of signal transduction	2440	26.6	61	6.57e-09
Negative regulation of response to stimulus	967	10.6	36	6.57e-09
Regulation of I-kappaB kinase/NF-kappaB cascade	210	2.29	17	1.55e-08
Cytokine mediated signaling pathway	374	4.08	21	7.53e-08
Negative regulation of biological process	4590	50.1	87	7.53e-08
I-kappaB kinase/NF-kappaB cascade	246	2.69	17	1.26e-07
Regulation of apoptotic process	1540	16.8	43	2.67e-07
Regulation of programmed cell death	1550	17	43	2.75e-07
Negative regulation of transcription, DNA-dependent	987	10.8	33	2.75e-07
Apoptotic process	2130	23.2	52	2.75e-07
Innate immune response	638	6.97	26	2.75e-07
Negative regulation of cellular biosynthetic process	1220	13.3	37	3.58e-07
Programmed cell death	2160	23.5	52	3.79e-07
Negative regulation of biosynthetic process	1240	13.6	37	4.96e-07
Negative regulation of nucleobase-containing compound metabolic process	1130	12.3	35	4.96e-07
Negative regulation of RNA metabolic process	1020	11.2	33	5.1e-07
Response to organic substance	2500	27.3	56	9.15e-07
Protein import into nucleus	228	2.49	15	9.82e-07
Nuclear import	232	2.53	15	1.19e-06
Interaction with host	426	4.65	19	6.26e-06
Regulation of defense response	519	5.67	21	6.69e-06
Nucleocytoplasmic transport	388	4.24	18	6.99e-06
Intracellular receptor mediated signaling pathway	270	2.95	15	7.47e-06
Nuclear transport	392	4.28	18	7.59e-06
Protein import	272	2.97	15	7.68e-06
Regulation of response to stimulus	3360	36.7	65	8.69e-06
Regulation of nucleocytoplasmic transport	172	1.88	12	9.82e-06
Regulation of intracellular transport	290	3.17	15	1.59e-05
Regulation of molecular function	2250	24.5	49	1.61e-05
Regulation of cytokine production	513	5.6	20	1.79e-05
Myeloid cell differentiation	296	3.23	15	1.89e-05
Organ development	3290	35.9	63	1.93e-05
Regulation of developmental process	1880	20.5	43	2.5e-05
Central nervous system development	784	8.56	25	2.56e-05
Cell surface receptor signaling pathway	3340	36.4	63	2.92e-05
Response to chemical stimulus	3830	41.8	69	3.69e-05
Negative regulation of transcription from RNA polymerase II promoter	552	6.03	20	4.65e-05
Regulation of transforming growth factor beta receptor signaling pathway	107	1.17	9	4.7e-05
Regulation of cell proliferation	1430	15.6	35	6.37e-05
Defense response	1510	16.5	36	8.1e-05
Intracellular protein kinase cascade	1140	12.4	30	8.22e-05
Negative regulation of cell proliferation	585	6.39	20	9.97e-05
Negative regulation of transferase activity	189	2.06	11	0.000119
Regulation of transcription from RNA polymerase II promoter	1610	17.6	37	0.000122
Protein targeting	545	5.95	19	0.000123
Regulation of cell differentiation	1290	14.1	32	0.000123
Positive regulation of developmental process	817	8.92	24	0.000132
Response to stress	4150	45.3	71	0.000132
Positive regulation of defense response	273	2.98	13	0.000136
Regulation of protein metabolic process	1820	19.9	40	0.000136
Positive regulation of signal transduction	998	10.9	27	0.000142
Regulation of multicellular organismal process	2480	27.1	49	0.000167
Regulation of immune system process	1190	13	30	0.000172
Positive regulation of transcription, DNA-dependent	1260	13.7	31	0.000173
Regulation of sequence-specific DNA binding transcription factor activity	372	4.06	15	0.000184
G1 phase	49	0.535	6	0.00019
Negative regulation of apoptotic process	679	7.41	21	0.000195
Negative regulation of phosphorylation	246	2.69	12	2,00E-04
Regulation of catalytic activity	1730	18.9	38	2,00E-04
Cytokine production	576	6.29	19	0.000203
Positive regulation of transcription from RNA polymerase II promoter	800	8.73	23	0.000223
Regulation of protein import into nucleus	137	1.5	9	0.000223
Negative regulation of phosphate metabolic process	293	3.2	13	0.000228
Negative regulation of programmed cell death	691	7.54	21	0.000229

Negative regulation of binding	78	0.852	7	0.000251
Immune response	1430	15.6	33	0.000262
Defense response to virus	215	2.35	11	0.000262
Cell proliferation	1900	20.8	40	0.000271
Multi-organism process	1710	18.6	37	0.000304
Regulation of myeloid cell differentiation	145	1.58	9	0.000315
Regulation of cell cycle	886	9.67	24	0.000336
Positive regulation of catalytic activity	1070	11.7	27	0.000345
Regulation of gene expression	4480	48.9	73	0.000373
Positive regulation of cytokine production	268	2.93	12	0.000379
Immune system development	722	7.88	21	0.000379
Positive regulation of RNA metabolic process	1330	14.6	31	0.000389
Intracellular signal transduction	2450	26.7	47	4,00E-04
Regulation of immune response	727	7.94	21	0.000404
Regulation of transcription, DNA-dependent	3770	41.2	64	0.000427
Regulation of nucleobase-containing compound metabolic process	4540	49.5	73	0.000519
Immune effector process	576	6.29	18	0.000519
Positive regulation of sequence-specific DNA binding transcription factor activity	198	2.16	10	0.000563
Immune system process	2720	29.7	50	0.000578
Cell cycle	1860	20.3	38	0.000694
Interphase of mitotic cell cycle	435	4.75	15	0.000723
Negative regulation of protein metabolic process	540	5.9	17	0.000732
Viral reproductive process	597	6.52	18	0.00077
Regulation of transferase activity	768	8.39	21	0.000778
Interphase	443	4.84	15	0.00085
Reproductive process	1740	19	36	0.00086
Positive regulation of metabolic process	2690	29.4	49	0.000867
Embryo development	1080	11.8	26	0.000907
Cell cycle process	1420	15.4	31	0.00097
Positive regulation of response to stimulus	1550	17	33	0.000997
Cellular response to stress	1620	17.7	34	0.00103
Positive regulation of nucleobase-containing compound metabolic process	1490	16.3	32	0.00103
Regulation of cellular protein metabolic process	1560	17	33	0.00103
Regulation of RNA metabolic process	3900	42.6	64	0.00106
Intracellular protein transport	793	8.66	21	0.00109
Transcription initiation from RNA polymerase II promoter	219	2.39	10	0.00109
Cell development	1840	20.1	37	0.00109
Hematopoietic or lymphoid organ development	679	7.41	19	0.00112
G1 phase of mitotic cell cycle	47	0.513	5	0.00112
Reproduction	1860	20.3	37	0.00118
Enzyme linked receptor protein signaling pathway	1180	12.8	27	0.00118
Negative regulation of cellular protein metabolic process	463	5.06	15	0.00118
Positive regulation of cell differentiation	571	6.23	17	0.00118
Multicellular organismal development	5720	62.4	85	0.00118
Cellular protein catabolic process	518	5.66	16	0.0012
Regulation of protein modification process	1250	13.6	28	0.00127

Extended Methods

Flow- and mass cytometry

Details of all antibodies used for flow cytometry are found in sTable1 and for mass cytometry in sTable2. Recombinant Human NKG2D Fc Chimera Protein (#1299-NK-050, R&D Systems) was biotinylated using the One-Step Antibody Biotinylation Kit (Miltenyi Biotec) as per manufacturer's recommendation. For all surface antibodies, cells were stained in PBS containing 2% FBS and 2 mM EDTA (Thermo Fisher, USA) for 15 min at room temperature followed by two washes. A second extracellular stain and incubation was performed with streptavidin and the pan-KIR binding antibodies KIR2DL2/S2/L3 and KIR2DL1/S1. After washing, cells were fixed for 15-20 min at 4 °C using BD Cytofix/Cytoperm™ Fixation and Permeabilization Solution (BD Biosciences). After two washes with BD Perm/Wash™ Buffer (BD Biosciences), intracellular markers were stained in this buffer for 30 min at room temperature. For HLA-C1/C2 expression, cells were stained with recombinant human KIR2DL1/CD158a or KIR2DL3/CD158b2-Fc Chimera Proteins (R&D systems) at 30 µg/ml in PBS 60 mins on ice, washed twice and incubated 15 mins at room temperature with Goat anti-Human IgG Fc Secondary Antibody, conjugated to PE. Samples were incubated with the secondary antibody only or isotype IgG1-PE as controls. Cells were fixed and acquired as described above. Flow cytometry samples were acquired on a BD LSRII or LSR Fortessa flow cytometer (BD Biosciences). Acquired data were analyzed in FlowJo v.10.6 (BD Biosciences) gated on live $CD19^{-}CD3^{-}CD56^{+}$ single cells or $CTV^{+}CD56^{-}$ target cells for cytotoxicity assays. AML blasts were defined by low CD45 expression. For visualization of flow cytometry data, t-Distributed Stochastic Neighbor Embedding (t-SNE), an unsupervised nonlinear dimensionality reduction algorithm, was employed in FlowJo software with default settings and included all fluorescent markers stained for. For mass cytometry experiments, cells were stained with Cell-ID Intercalator-103Rh (Fluidigm) for viability assessment followed by an Fc

blocking reagent and a surface antibody cocktail. Antibodies that were not available pre-labelled were conjugated to metal isotopes using Maxpar X8 Antibody Labeling Kits (Fluidigm). Cells were fixed with 2% formaldehyde in Maxpar PBS (Fluidigm). Samples were washed, permeabilized and barcoded using the Cell-ID 20-Plex Pd Barcoding Kit (Fluidigm) before being pooled. Samples were then resuspended in cold methanol and stored overnight at -20 °C. Upon acquisition, cells were stained with an intracellular antibody cocktail, labeled with Cell-ID Intercalator-Ir (Fluidigm) and resuspended in Maxpar Cell Acquisition Solution (Fluidigm). EQ Four Element Calibration Beads (Fluidigm) were added to the cells before analysis on a Helios CyTOF instrument (Fluidigm). FCS files were concatenated, normalized and debarcoded using Helios software (Fluidigm), and gated using FlowJo v.10 (BD Biosciences). For subsequent analysis, data was imported into R (R Core Team, 2019) using the flowCore package, and transformed using arcsinh(x/5). For mass-cytometry data, t-SNE was performed using the Rtsne R package with default settings and results were visualized using the ggplot2 R package.

Cellular indexing of transcriptomes and epitopes by sequencing - CITE-Seq.

CITE-seq was performed as outlined in Biolegend 'TotalSeqTM-A Antibodies and Cell Hashing with 10x Single Cell 3' Reagent Kit v3 3.1 Protocol' with minor modifications. Briefly, cells were stained with CD56-biotin (REA 196), followed by TotalSeq antibodies and TotalSeq streptavidin-PE and Live/Dead Aqua (Invitrogen) and subsequently sorted for viable $CD56^+$ cells by flow cytometry. Day 0 cells from the same donor were thawed and recovered in IL-15 (3 ng/ml, Miltenyi) overnight prior to staining and sorting. To distinguish day 0 and day 11 cells, streptavidin-PE conjugated to different oligos were used, and equal number of sorted viable $CD56^+$ cells from the two cell preparations were pooled as one sample. This was followed by standard 10X Genomics library preparation and sequencing workflow (Genomics

Core Facility UiO, Oslo, Norway). Sequencing was performed with the recommended read lengths on the NextSeq500 sequencer (Illumina). Data were demultiplexed using bcl2fastq2 (Illumina) and further processed with the Cell Ranger version 6.0.1 software package to generate raw count-matrix files. The antibody-derived tags (ADTs) were counted using the package CITE-Seq-Count[2]. CITE-Seq data was analyzed using Scanpy[3] as follows: During pre-processing cells were filtered out based on number of genes and reads in each cell (minimum 300 genes, maximum 30 000 total counts) and genes were filtered based on number of cells in which the genes were expressed (minimum 3 cells). Cells with more than 10% mitochondrial reads were also filtered out. Cells with expression of CD3 were filtered out together with cells expressing both day 0 and day 11 ADTs. The remaining cells (n=8013) were analyzed using the probabilistic model totalVI[4]. This model was used to get a representation of the cells that were used for clustering and for embedding using UMAP, get denoised expression values for both the ADTs and mRNA, and to perform the statistical tests to identify differentially expressed (DE) genes. Heatmaps, UMAP plots, violin plots and dot plots were generated using Scanpy[3]. The p-values and fold change computed from the DE framework in totalVI were plotted as volcano plots using bioinfokit[5]. The genes down- and upregulated in CAR T cells after continuous antigen exposure were extracted from Good et al.[1] and for both gene sets the overlap with the genes upregulated in the day 11 CITE-Seq cluster was shown in Venn diagrams. AUCell[6] was used to identify the activity of a T cell-derived exhaustion signature from Feucht et al.[7] in the CITE-Seq data and the AUC scores were plotted as violin plots showing the activity of this signature in the various clusters.

Tumor cell lines

The K562 cell line (chronic myeloid leukemia) and 721.221 (EBV-transformed B cell line) were obtained from ATCC. HL-60 (acute myeloid leukemia) was obtained from the Miller lab.

PANC-1 (Pancreatic ductal adenocarcinoma) and A549 (lung adenocarcinoma) were obtained from Fate Therapeutics. K562, A549 and PANC-1 along with NALM-6 (B cell acute lymphoblastic leukemia) and BJAB (Burkitt's Lymphoma) were re-authenticated using STR fingerprinting (ATCC). The K562, 721.221, BJAB and NALM-6 cell lines were cultured in RPMI-1640 (Thermo Fisher) with 10% FBS and 1 mM L-glutamine (Thermo Fisher) ('complete RPMI') and with or without 100 mg/ml of penicillin/streptomycin (Invitrogen). HL-60 was grown in IMDM, A549 and PANC-1 in DMEM, all with 10% FBS and with or without 100 mg/ml of penicillin/streptomycin (Invitrogen). Cell lines were KIR-ligand genotyped with PCR-based KIR-HLA Ligand kit (Olerup SSP). All cell lines were mycoplasma-tested using one of the following reagents: MycoAlert^{TM} PLUS Mycoplasma Detection Kit (Lonza), the PCR-based LookOut Mycoplasma (Sigma Aldrich), or Mycoplasma check (Eurofins Genomics).

Flow cytometry-based NK cell functional assays

NK cell degranulation and cytokine production was evaluated by mixing ADAPT-NK products at different E:T ratios with tumor targets and incubating in complete RPMI in 96-well U-bottom plates for 6 h at 37 °C and 5% CO_2. Monensin (GolgiStop, 1:1500, BD Biosciences), Brefeldin A (GolgiPlug, 1:1000, BD Biosciences) and anti-CD107a were added 1 h into the 6 h co-culture. To measure ADCC, MabThera (1 μg/ml) was added to co-culture experiments with 721.221 cells. Phorbol-12-myristate-13-acetate (PMA) (50 ng/ml) + ionomycin (1 μg/ml) (Sigma) was used as positive control. NK cell cytotoxicity was evaluated by mixing ADAPT-NK products at different E:T ratios with single target cell lines pre-stained with 2 μM of CellTrace Violet (Thermo Fisher) and incubating in 96-well V-bottomed plates for 6 h at 37 °C and 5% CO_2. FITC-DEVD-FMK (Abcam), a caspase-3 inhibitor used to detect the active/cleaved form of Caspase-3 was added at the start of the incubation and cells were

subsequently stained with eFluor780 Fixable viability dye (Thermo Fisher). All samples were fixed and acquired on a flow cytometer as described above. Dead cells were defined as CellTrace^{+} Caspase-3^{+} and/or dead cell marker (DCM)$^{+}$ and specific cytotoxicity calculated as: ($\%\ dead^{\ experimental}$- $\%\ dead^{\ target\ only}$) ÷ (100% - $\%\ dead^{\ target\ only}$) x 100%. For competitive cytotoxicity assays, HLA-C1- and HLA-C2-dimer expressing K562 were stained with either high (2 µM) or low (0.5 µM) concentrations of CellTrace Violet respectively, prior to being mixed at a 1:1 ratio and subsequently seeded at multiple E:T ratios with ADAPT-NK cells. The two different target cells were then identified based on CellTrace^{+} high or low, and in these populations the frequency of live (Caspase-3^{-} and/or DCM^{-}) remaining in the culture out of the total live CTV^{+} cells was determined. Cytokine stimulations with IL-12 (10 ng/ml) and IL-18 (10 ng/ml) (both Biotechne) were performed for 25 h at 37 °C and 5% CO_2, and Monensin and Brefeldin A were added after 20 h.

Genetic cell engineering

In brief, K562 cells (ATCC Cat: CCL-243) were engineered by Fate Therapeutics using an in-house third-generation lentiviral transfer plasmid designed to contain a chimeric protein with the HLA-G leader peptide (1-24) and the mature HLA-E*0103 (22-358) driven by an EF1α promoter. Lentivirus was produced in Lenti-X™ 293T cells (Clontech 632180) using the second-generation packaging system that includes a transfer plasmid, a packaging plasmid, for expression of gag, pol, tat and rev, and an envelope plasmid, for expression of envelope protein VSV-G. The transduced population was sorted to enrich for cells expressing high levels of HLA-E. For HLA-C and -E variants, K562 were engineered in-house using VSV-G-pseudotyped lentiviral particles with a mammalian LeGO-G2 expression vector, to express the following synthetic proteins: β2m–HLA-C1 single chain dimer (HLA-C1-dimer): *B2M* signal peptide sequence and mature *B2M* (without STOP-codon) fused covalently to mature *HLA-*

*C*07:01* (without STOP-codon) through a flexible $(G_4S)_4$ linker sequence, followed by a T2A peptide sequence and murine *Thy1.1* (*CD90.1*) ; β2m–HLA-C2 singe chain dimer (HLA-C2-dimer): *B2M* signal peptide sequence and mature *B2M* (without STOP-codon) fused covalently to mature *HLA-C*04:01* (without STOP-codon) through a flexible a $(G_4S)_4$ linker sequence, followed by a T2A peptide sequence and murine *Thy1.2* (*CD90.2*); HLA-G_{3-11}–β2m–HLA-E single chain trimer (HLA-E-trimer): *B2M* signal peptide sequence and the *HLA-G*$_{3-11}$ signal peptide sequence (VMAPRTLFL) fused covalently by a flexible $(G_4S)_3$ linker sequence to mature *B2M* (without STOP-codon), which is further covalently linked to mature *HLA-E*01:01* by a flexible $(G_4S)_4$ linker sequence. Expression cassettes were sub-cloned into the mammalian expression vector LeGO-G2 (kind gift from Boris Fehse, Addgene plasmid #251917; http://n2t.net/addgene:25917; RRID: Addgene_25917) after removing EGFP. LeGO-HLA-C1-dimer, LeGO-HLA-C2-dimer, and LeGO-HLA-E-trimer were used to generate VSV-G-pseudotyped lentiviral particles. For production of VSV-G-pseudotyped lentiviral particles, 22 x 10^6 LentiX 293T cells (Takara) were plated into T125 flask one day prior to transfection with 15 µg of LeGO expression vector, 10 µg pRSV-Rev (Addgene plasmid #12253), 15 µg pMDLg/pRRE (Addgene plasmid #12251) and 5 µg pCMV-VSV-G (Addgene plasmid #8454) using Lipofectamine 3000 (Invitrogen). Supernatant containing lentiviral particles was harvested at 24 and 48 h post transfection and concentrated using LentiX concentrator (Takara). Viral titers were determined using Lenti-X GoStix (Takara). Concentrated virus was stored as aliquots at -80 °C until use. Wildtype K562 cells were then transduced with 20 MOI of lentiviral particles at 2,5 x10^5 / 24-well in a total volume of 1 ml. The plates were centrifuged at 900 *g* for 1 h at 32 °C followed by incubation at 37 °C overnight. Media was exchanged the next day and cells were cultivated for >5 passages. After >5 passages, genetically engineered K562 lines were sorted on a MA900 cell sorter (Sony) for high HLA-C-dimer expression using recombinant human KIR2DL3 Fc chimera proteins and

KIR2DL3 Fc chimera proteins, respectively or sorted for high HLA-E-trimer expression using an anti-HLA-E antibody (3D12, BioLegend). Sorted cells were routinely monitored for HLA-C-dimer and HLA-E-trimer expression and tested for presence of mycoplasma (Eurofins Mycoplasma check). Further, NALM-6 were engineered to knock-out HLA-E using Cas9 and a pool of synthetic guide RNAs (sgRNA) (5'- AUUUCCACACUUCCGUGUCC-3', 5'-ACAACGACGCCGCGAGUCCG-3' and 5'-GGGGUCAGAGUAUUGGGACC-3') (CRISPRevolution sgRNA EZ Kit, Synthego). 180 pmol sgRNA and 20 pmol Cas9 (Synthego) were used to form ribonucleoprotein (RNP) complexes. 1.5 x 10^5 cells in 3P buffer (5 mM KCl, 15 mM MgCl2, 90 mM NaCl, 10 mM Glucose, 0.4 mM Ca(NO3)2, 40 mM Na2HPO4/NaH2PO4 pH7.2) were mixed with RNP and electroporated using Lonza 4D-Nucleofector system with pulse code CV-104. Cells were grown for 7 days before single cells were sorted on a FACS Aria II (BD Biosciences) to establish multiple clones in 96-well round bottom plates. DNA sequencing and flow cytometry confirmed HLA-E depletion in the selected clones used in the study. HLA-E sgRNAs further targeted the HLA-C locus resulting in a combined KO for this line.

Serial killing assays

The cytotoxic potential of individual NK cells was evaluated using a previously described microwell chip screening assay[8]. Briefly, NK cells labelled with 5 µg/ml CellTrace Yellow and target cells labelled with 5 µg/ml CellTrace Far Red (Thermo Fisher) were seeded in a microwell chip containing complete RPMI supplemented with 100 ng/ml Sytox Green (Thermo Fisher). Cells stochastically distributed in the 8064 60-µm-wide wells at an average effector to target ratio of 1:5. HLA-C matched and -mismatched co-cultures with the same ADAPT-NK product were investigated simultaneously in different compartments of the chip. The co-cultures were imaged every 3 h for 15 h using an inverted confocal microscope, LSM 880 (Carl

Zeiss AG) equipped with a 10x/0.5 Plan-Achromat objective and an environmental control unit (37ºC, CO_2 5%). The images were processed using a custom-built MATLAB script whereby live and dead cells were quantified at all time points. For each condition, a minimum of 500 wells containing a single NK cell at start along with at least 4 live target cells were included for analysis. Target cell death was adjusted for spontaneous death using wells containing only target cells.

IncuCyte measurement of tumor killing

Tumor killing was measured in real time using the IncuCyte® S3 platform. Target cells stably expressing NucLight Red (Essen Biosciences) were selected for NucLight Red expression after thawing with 2 µg/mL puromycin for 72 h, screened by flow cytometry for uniform NucLight Red expression and allowed to rest without puromycin for at least 48 h (1 passage) before assay. Target cells were seeded at 5000 cells/well in 200 uL of complete media and allowed to attach overnight in clear, 96-well flat-bottom plates (Thermo Fisher, cat. #167008). Wells used for target cells which habitually grow in suspension (HL60, K562, Bjab, Nalm6) were pre-coated with Poly-L-Ornithine solution (Sigma/Merck, cat #A-004-M). At assay start half of the media was removed and ADAPT-NK cells were serially diluted and added to the wells in SCGM (CellGenix) with 50 IU/mL IL-2 at different E:T ratios. Images (3/well) from at least two technical replicates for each condition were acquired every 90 min for 48 h, using a 10x objective lens and analyzed by IncuCyte Controller v2020A (Essen Biosciences). Graphed readouts represent percentage live target cells (NucLight Red$^+$). To obtain percentage of live cells, live target cell count in each well was normalized by division to timepoint 0 h (T0), and then to the number of live cells in the "target cell only" control group, as calculated in the following equation: % Live cells = [(Tn/T0) test / (Tn/T0) ctrl] x 100. The means of the technical replicates for each condition were compiled for n=4-9 donors, tested in at least two

independent experiments for each target cell, and are displayed as mean (± SD). Area Under Curve for each condition was graphed and analyzed.

In vivo AML tumor model

Fifteen male 9-11 weeks old NOD.Cg-PrkdcscidIl2rgtm1Wjl/SzJ mice (NSG, Jackson Laboratories) mice were injected intravenously with HL-60 cells stably expressing firefly luciferase (1.5x10^6/mouse). Four control mice on the same background received no injections. After allowing tumors to engraft for 4 days, bioluminescence imaging was performed, and mice were randomized into 3 groups. Mice in the first group received no treatment (tumor alone). Mice in the second group received intravenous injections of HLA-C/KIR matched ADAPT-NK cells (flat-dose 5 x10^6/mouse) expanded as above. Mice in the third group received intravenous injections of HLA-C/KIR mismatched ADAPT-NK cells (flat-dose 5x10^6/mouse). Mice receiving NK cell injections were also injected intraperitoneally with IL-15 (6ug/mouse, National Cancer Institute) twice weekly for three weeks. Bioluminescence imaging was performed weekly to track tumor burden using the IVIS Spectrum (Perkin-Elmer). Images were analyzed using Living Image software (Perkin-Elmer). D-Luciferin Sodium Salt (GoldBio) was dissolved into DPBS at a concentration of 30 mg/ml and injected intraperitoneally at a dose of 3 mg/20 g body weight.

References extended methods

1. Good, C.R. *et al.* An NK-like CAR T cell transition in CAR T cell dysfunction. *Cell* **184**, 6081-6100 e6026 (2021).

2. Gui, P.R.b.B.F.s.G. Hoohm/CITE-seq-Count: 1.4.2. Zenodo; 2019.

3. Wolf, F.A., Angerer, P. & Theis, F.J. SCANPY: large-scale single-cell gene expression data analysis. *Genome Biol* **19**, 15 (2018).

4. Gayoso, A. *et al.* Joint probabilistic modeling of single-cell multi-omic data with totalVI. *Nat Methods* **18**, 272-282 (2021).

5. Bedre, R. reneshbedre/bioinfokit: Bioinformatics data analysis and visualization toolkit. . Zenodo; 2021.

6. Aibar, S. *et al.* SCENIC: single-cell regulatory network inference and clustering. *Nat Methods* **14**, 1083-1086 (2017).

7. Feucht, J. *et al.* Calibration of CAR activation potential directs alternative T cell fates and therapeutic potency. *Nat Med* **25**, 82-88 (2019).

8. Guldevall, K. *et al.* Microchip Screening Platform for Single Cell Assessment of NK Cell Cytotoxicity. *Front Immunol* **7**, 119 (2016).
