# Supplementary material for: Adaptive single-KIR+NKG2C+ NK cells expanded from select superdonors show potent missing-self reactivity and efficiently control HLA-mismatched acute myeloid leukemia
Source: J Immunother Cancer. 2022 Nov 1;10(11):e005577. doi: 10.1136/jitc-2022-005577 (PMC9628692; doi:10.1136/jitc-2022-005577)
Supplement: Supplementary data [file jitc-2022-005577supp002.pdf]

Adaptive single-KIR$^+$NKG2C$^+$ NK cells expanded from select superdonors show potent missing-self reactivity and efficiently control HLA-mismatched acute myeloid leukemia

Authors:
A. Haroun-Izquierdo, M. Vincenti, H. Netskar, H. van Ooijen, B. Zhang, L. Bendzick, M. Kanaya, P. Momayyezi, S. Li, M. Thune Wiiger, H. J. Hoel, S. Zandstra Krokeide, V. Kremer, G. Tjønnfjord, S. Berggren, K. Wikström, P. Blomberg, E. Alici, M. Felices, B. Onfelt, P. Hoglund, B. Valamehr, H.G. Ljunggren, A. Bjorklund, Q. Hammer, L. Kveberg, F. Cichocki, J. S. Miller, K.-J. Malmberg* and E. Sohlberg*

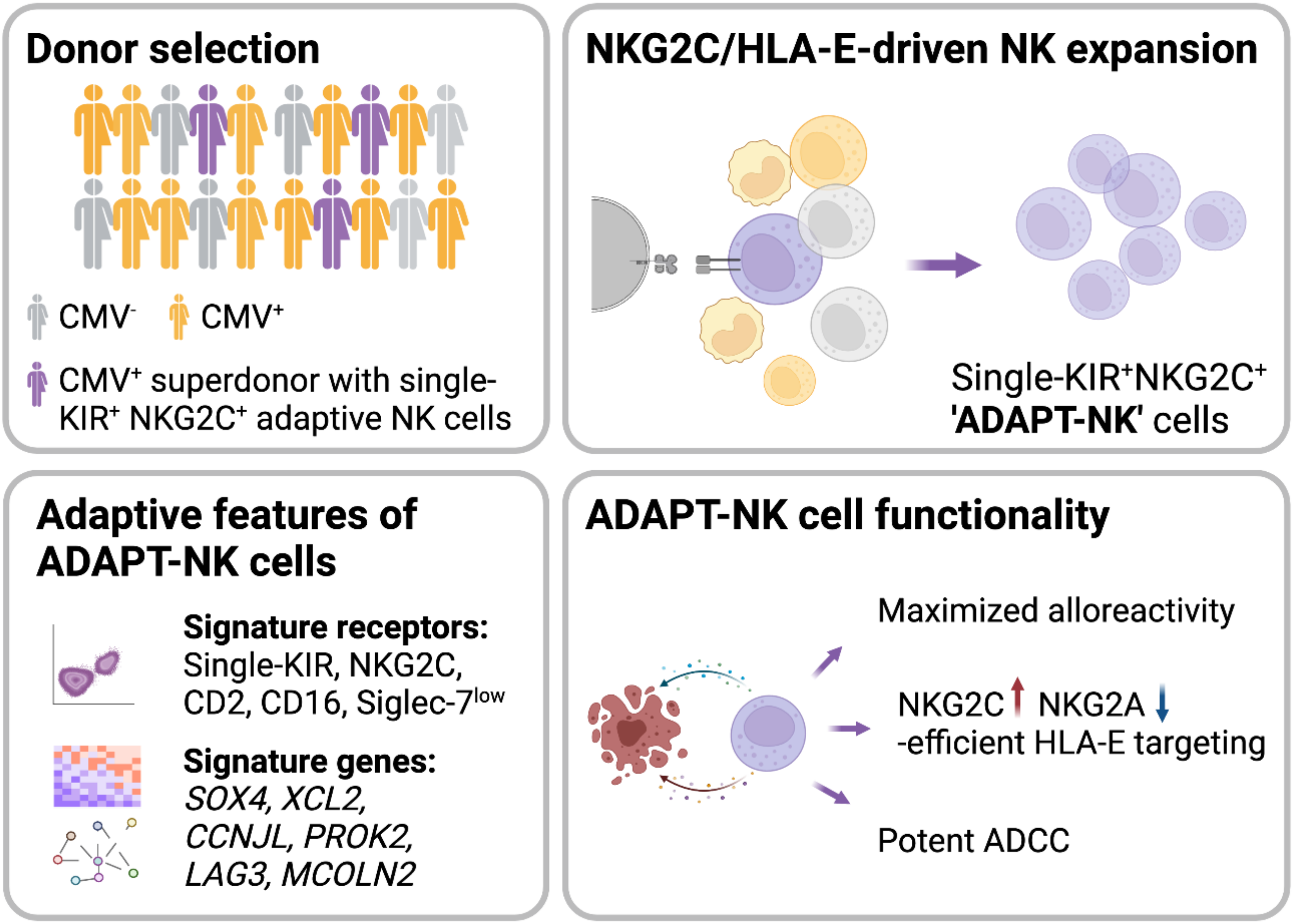

In brief:

1. Novel GMP-compliant protocol to expand single self-KIR$^+$ adaptive NK cells from third-party 'superdonors'.
2. Strong alloreactivity in a mouse model of AML as well as against primary AML blasts.
3. ADAPT-NK cells overcome the HLA-E checkpoint and display potent ADCC
